# Hydrogel-based systems with nanoparticle and microencapsulation for food preservation: Preparation, characterization, and application

**DOI:** 10.1016/j.fochx.2026.103489

**Published:** 2026-01-06

**Authors:** Hui Zhou, Wenying Yuan, Chenfeng Xu, Xuan Huang, Weitao Zhao, Zhen Wu, Daodong Pan, Jie Luo, Lei Zhou, Xiankang Fan

**Affiliations:** a College of Food Science and Technology, Hunan Agricultural University, Changsha, Hunan 410128, China; b Changsha Modern Food Research Institute, Hunan Agricultural University, Changsha, China; c Key Laboratory of Animal Protein Food Processing Technology of Zhejiang Province, College of Food and Pharmaceutical Sciences, Ningbo University, Ningbo 315832, China

**Keywords:** Hydrogel, Food preservation, Nanoparticle composite, Microencapsulation, Active packaging, Shelf-life extension

## Abstract

Global food spoilage, driven by microbial growth and oxidative deterioration, necessitates advanced preservation strategies. This study systematically reviews the preparation methods, structural and functional characterization, and applications of these hybrid systems across dairy, fruit, and meat products. The incorporation of nanoparticles (e.g., Ag, Cu, melanin NPs) and microencapsulated bioactive agents (antioxidants, probiotics, antimicrobials) into hydrogel matrices significantly enhances mechanical stability, antibacterial efficacy, humidity regulation, and stimuli-responsive release capabilities. This synergy arises from three complementary mechanisms, including nanoparticles that reinforce the hydrogel network and provide potent antimicrobial activity, microencapsulations that offer robust protection and controlled release of sensitive bioactives, and the hydrogel itself serves as a responsive scaffold for moisture management and targeted delivery. This synergistic function has excellent applications in probiotic protection, intelligent freshness monitoring of fruits, and odor control of meats. This study underscores the transformative potential of hydrogel-nanoparticle-microcapsule systems in advancing active and intelligent food packaging.

## Highlights

• Hydrogel systems extend food shelf-life via nanoparticles and microcapsules.
• The combination boost hydrogel stability and responsiveness.
• Effective in dairy, fruits, and meat preservation.
• Future focus on scalable synthesis and real-time monitoring.

## Graphical abstract

**Figure:**
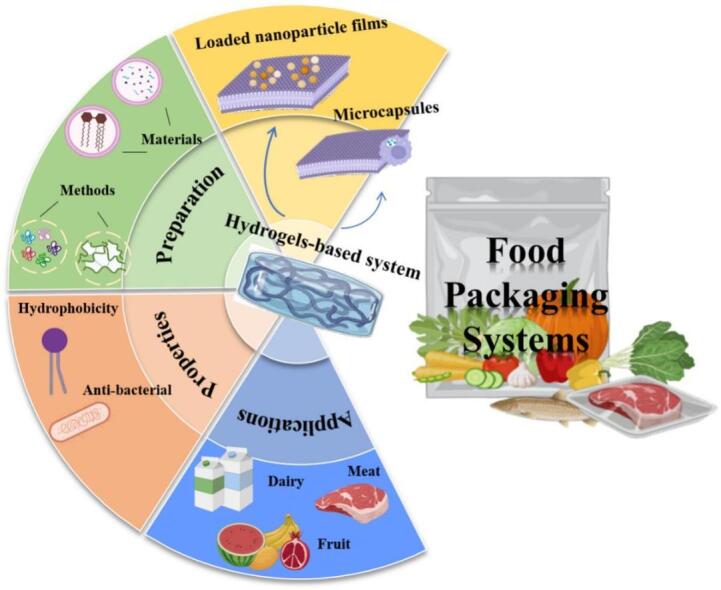

## 1 Introduction

Food spoilage is a significant global challenge, causing an annual waste of approximately 1.3 billion tons and posing a direct threat to food safety and public health (Xu et al., 2025). This process is primarily driven by microbial contamination (e.g., bacteria and fungi) and oxidative deterioration (including lipid oxidation, nutrient degradation, and deterioration of color and flavor) (Zhou, Zhang, & Wang, 2022). Although traditional preservation methods, such as refrigeration, chemical preservatives, and controlled atmosphere packaging, have been widely used, they face substantial limitations. Refrigeration, for instance, consumes significant amounts of energy and fails to inhibit the growth of psychrophilic bacteria fully. Chemical preservatives may raise health concerns among consumers and are subject to regulatory restrictions. Moreover, passive packaging, such as ordinary plastic, only provides a physical barrier and cannot actively address spoilage factors caused by internal or environmental changes (Ma et al., 2022). Given these limitations, the development of more efficient, safe, intelligent, and sustainable food preservation technologies is urgently needed.

Hydrogels, with their unique three-dimensional hydrophilic polymer network structure, have emerged as promising candidates for overcoming traditional food preservation barriers. Their core advantage lies in their ability to simulate biological tissue environments, effectively maintaining the moisture content of food (especially fresh fruits and vegetables), reducing weight loss and wilting, and thereby preserving freshness and texture (Du et al., 2025). Furthermore, commonly used natural polymer materials, such as chitosan, sodium alginate, and carboxymethyl cellulose, are safe, non-toxic, and biodegradable, meeting food contact requirements while reducing environmental burdens (Zhang, Zhao, et al., 2023). Hydrogels can also efficiently encapsulate and protect various bioactive substances, including antibacterial agents, antioxidants, probiotics, enzymes, and nutrients. They are sensitive to environmental changes such as pH, temperature, humidity, specific enzymes, or chemicals, enabling intelligent targeted release of active ingredients (Merino et al., 2022). Additionally, hydrogels can be easily processed into films, coatings, microspheres, or monolithic gels to suit different food types and packaging needs. These properties have led to successful applications in real-time safety monitoring (e.g., indicator labels), bioactive substance delivery (e.g., probiotic protection), and their use as direct active or smart packaging materials (Karakus et al., 2024). However, a critical gap persists between the potential of single-component hydrogels and the complex demands of real-world food preservation. Pure hydrogel systems often suffer from inherent trade-offs: high water content can compromise mechanical integrity, limiting their durability in packaging, while their functionality is often restricted to a single mode of action (e.g., providing only moisturizing or antibacterial effects). The intricate challenge of preserving diverse food matrices that each have unique spoilage pathways requires a more sophisticated, multi-functional approach. This necessity has catalyzed a paradigm shift from single-component systems to integrated, hybrid material platforms.

It is within this context that this review critically examines the strategic integration of hydrogels with nanoparticles and microcapsules. This synergistic system overcomes the intrinsic limitations of individual components by addressing the mechanical weakness of hydrogels, the functional constraints of isolated nanoparticles, and the inadequate barrier properties and controlled release capabilities of microcapsules. Specifically, embedding nanoparticles (NPs, 1–100 nm) of metals (e.g., Ag, Cu), metal oxides (e.g., TiO₂, ZnO), organic materials (e.g., liposomes, protein nanoparticles), or natural origin materials (e.g., melanin nanoparticles) into hydrogel matrices or loading them onto hydrogel films has emerged as a promising innovation. These nanoparticles significantly enhance the system's mechanical properties through interfacial interactions, boost antibacterial and antioxidant activities owing to their high specific surface area and quantum effects, improve barrier properties such as resistance to water and oxygen, and provide unique photothermal/thermal functions (e.g., photothermal antibacterial activity, UV shielding). For example, Cu-loaded halloysite nanotubes (HNTs-Cu) enhance the hydrogel network via hydrogen bonding, significantly improving the composite membrane's hydrophobicity and thermal stability (Zhao et al., 2024). Similarly, chitosan membranes loaded with curcumin-zein-quercetin-chondroitin sulfate nanoparticles have been used for intelligent monitoring of fish freshness (Zhang, Yu, et al., 2024).

Nanoencapsulation technology (10–1000 nm) operates at an intermediate scale, enveloping bioactive agents within nanocarriers such as nanoliposomes (Lopez-Polo et al., 2021), solid lipid nanoparticles (Pink et al., 2019), and biopolymer-based nanocapsules (Liu, Liang, et al., 2023). This approach improves protection against environmental stressors. It can increase bioavailability and enable controlled release kinetics. These effects are stronger than those of free compounds, but they differ from microscale delivery systems. When incorporated into hydrogel networks, nanoencapsulated ingredients achieve synergistic stability and targeted functionality. For example, Huang et al. (2025) present a novel approach for enhancing the survivability of *Lactiplantibacillus plantarum* BXM2 using bamboo shoot-derived nanocellulose hydrogels.

Microencapsulation technology (1–100 μm) provides a physicochemical barrier for easily degradable active ingredients, such as vitamin E, polyphenols, probiotics, flavor substances, and essential oils. In microcapsule systems, hydrogels can serve as the core, wall material, or embedding matrix. This combination effectively protects active ingredients during processing and storage and enables precise controlled release during digestion or at target sites, thereby significantly improving bioavailability and functional effects. For example, smart-responsive microencapsulation hydrogels prepared by Pickering emulsion polymerization can respond to multiple stimuli and achieve precise release (Zhang et al., 2019). Additionally, curcumin/gum arabic/gelatin microcapsules embedded in alginate gel beads optimize curcumin release efficiency in the intestine (Lin et al., 2024).

Hydrogels, nanoparticle-loaded membranes, and microcapsules form a complementary and often integrated ternary system. Hydrogels serve as flexible, moisturizing, and responsive scaffolds; nanoparticles enhance the barrier performance and mechanical strength of hydrogel membranes, while providing antibacterial and ultraviolet protection; microcapsules offer robust protection and targeted release of sensitive active agents. This integration creates a powerful platform that simultaneously enables multiple protections, intelligent sensing and response, and precision-controlled release, thereby significantly extending food shelf life.

Although hydrogel-based systems have shown great potential in the field of food preservation, there is still a lack of a systematic summary, in-depth mechanism analysis, and comprehensive comment on the future prospects of the hydrogel-nanoparticle-microcapsule synergistic system. The purpose of this review is to provide an in-depth understanding of the preparation methods of hydrogel composites, detailing their key structural and functional properties and their correlation with performance. It systematically examines the application, fresh-keeping mechanism and practical effect of these integrated systems in key food fields such as dairy products, fruits, vegetables and meat products. Finally, this review will identify current challenges and indicate future research directions.

## 2 Hydrogel-based systems

### 2.1 Hydrogel

**Fig. 1:**
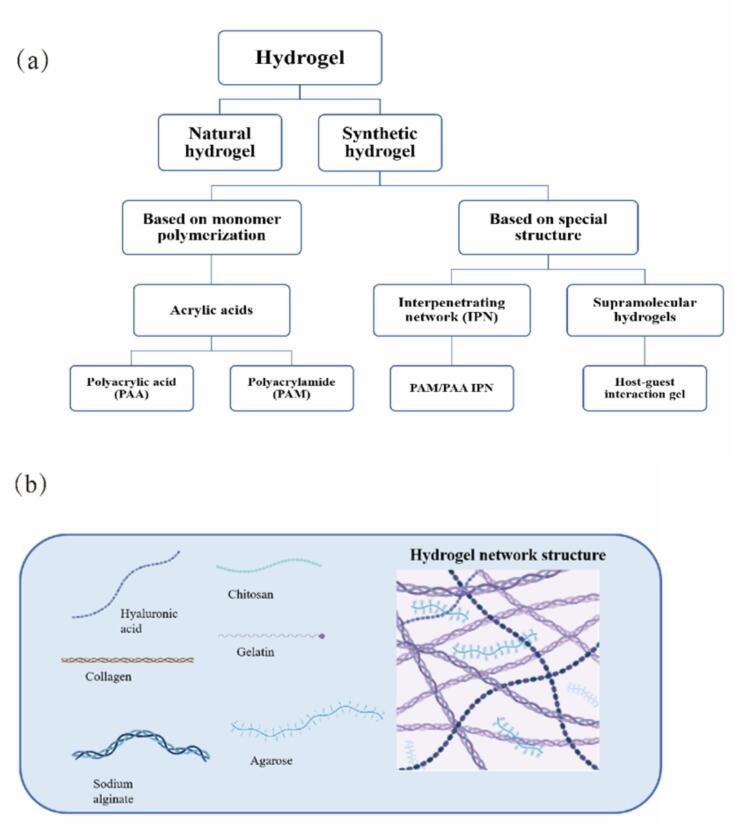
Hydrogels are classified into two major categories: natural hydrogels and synthetic hydrogels.

Hydrogels are polymeric materials characterized by three-dimensional cross-linked molecular networks that can absorb and retain significant amounts of water. The network formation occurs through either covalent or non-covalent interactions, such as physical entanglement and hydrogen bonding (Yang, 2022). The ability to engineer a diverse range of physicochemical properties such as elasticity, adhesion, and self-healing capacity, has enabled hydrogels to be widely applied across various fields. Natural hydrogels and synthetic hydrogels differ in their sources, chemical composition, physical properties, and application fields. Natural polymer materials are mainly derived from biological macromolecules in nature, such as hyaluronic acid, collagen, sodium alginate, chitosan, gelatin, agarose, etc. As shown in Fig. 1, synthetic hydrogels can be further divided into two major categories: those based on monomer polymerization and those based on special structures. Hydrogels based on monomer polymerization are primarily prepared through the polymerization of monomers such as acrylic acid, including polyacrylic acid (PAA) and polyacrylamide (PAM). Hydrogels with special structures, on the other hand, include interpenetrating networks (IPNs) and supramolecular hydrogels. Such hydrogels are formed by unique molecular arrangements and cross-linking methods, which confer distinct physicochemical properties.

In medicine, hydrogels are engineered to mimic natural tissues and boost stem cell production for soft tissue engineering. They also exhibit excellent properties for motion detection and information transmission, as well as enabling controlled drug release in delivery systems through environmental responsiveness (Chuang et al., 2018; Sun et al., 2020;Zhang, Yao, & Raffa, 2024). In food science, materials like chitosan, sodium alginate, and carboxymethyl cellulose are frequently used as substrates to prepare composite hydrogel films due to their biocompatibility, biodegradability, potential for chemical modification, and antibacterial properties (El Sayed, 2023). Lan et al. (2018) developed a calcium chloride-crosslinked carboxymethyl cellulose/sodium alginate/chitosan film. Its high tensile strength directly enhances packaging resistance to mechanical stress. This reduces film rupture and limits microbial ingress, which can extend shelf life. Its optimized water vapor transmission rate further regulates moisture balance, inhibiting both dehydration and moisture-induced spoilage. Similarly, Mohammed et al. (2023) fabricated edible coatings from nano-chitosan/nano-alginate hybrids. In these systems, the nano-scaled structure amplifies antibacterial efficacy. This is achieved by enhancing disruption of the bacterial cell membrane, which directly suppresses pathogen proliferation on food surfaces and thus reduces spoilage rates.

Various hydrogel preparation methods (Table 1) achieve tailored properties. Chemical crosslinking builds mechanical strength and long-term stability to prevent packaging failure; stepwise polymerization enables precise adjustability of antimicrobial release kinetics; radiation polymerization preserves bioactive compounds under mild conditions; solution polymerization offers simple, scalable control over film uniformity; and physical crosslinking eliminates chemical residues for safe edible applications. Common materials used in hydrogel preparation include polyester, polyamide, and other polymers, which demonstrates the broad applicability of hydrogels. To further enhance the biocompatibility, degradability, and environmental safety of hydrogels, ongoing research is focused on finding novel, environmentally friendly crosslinking agents. For instance, enzymatic crosslinking and click chemistry are employed to prepare hydrogels, while naturally derived polymers are used to create hydrogels with excellent biocompatibility and biodegradability (Guo et al., 2020). These approaches aim to reduce the environmental and health risks linked to conventional chemical cross-linkers. They also broaden potential applications. With further research, more environmentally friendly and efficient crosslinkers may be developed in the future.

**Table 1:** Main preparation methods of hydrogel-based membranes.

Classification	Preparation method	Advantage	Examples	Ref.
Hydrogel films	Chemical crosslinking	Good stability and suitable for long-term use	Pectin/acrylamide	(Siddiqua et al., 2021)
	Free radical polymerization	Rapid polymerization	Chitosan	(Tian et al., 2021)
	Stepwise polymerization	High adjustability	β-cyclodextrin	(Noor et al., 2023)
	Solution polymerization	Simple operation, easy to control	Chitosan/lysine/gelatin	(Li et al., 2025)
	Radiation polymerization	Under mild conditions	ZnO-NRs/cellulose/starch	(Chen et al., 2023)
	Physical crosslinking	No use of chemical reagents	Sodium alginate/chitosan/zinc ion	(Shi et al., 2022)
Nanoparticle loaded hydrogel films	Sol-gel process	Easy to control	ZnO-chitosan nanoparticles/ modified starch matrix	(Hu et al., 2019)
	Situ synthesis-loading method	Make nanoparticles highly fragmented and uniform distribution	Silver nanoparticles/dialdehyde cellulose	(Du et al., 2022)
	Layer-by-layer self-assembly method	Precise control, large area preparation, versatility	Konjac glucomannan/zein/tannic acid nanoparticles	(Liu et al., 2024)
	Electrospinning	Prepare films with high porosity and large specific surface area	Polycaprolactone/chitosan/Chinese yam polysaccharide	(Zang et al., 2024)
	Layer-by-layer self-assembly method	No chemical reagent，suitable for food industry, biocompatible	Chitosan/urea-formaldehyde resin	(Liu et al., 2024)
	Yeast microcapsule method	Safe, non-toxic, targeted and easy to obtain	Wheat germ oil/ soybean protein isolate/maltodextrin	(Cetinkaya et al., 2021)
Hydrogel-based microcapsule films	Spray drying method	Suitable for mass production, heat sensitive substances	Gum Arabic/ maltodextrin/whey protein isolate/inulin/sucrose	(Wang & Mutukumira, 2022)
	Sol-gel process	High specific surface area and high porosity	Janus silica nanoparticles/ AP-g-PNIPAAM	(Shi et al., 2023)
	Complex condensation method	Easy to adjust the particle size and surface properties of microcapsules	Black pepper essential oils/faba bean protein /chia seed polysaccharides	(Napiorkowska et al., 2024)
	Interfacial polymerization	Suitable for water-insoluble core material	Osmanthus essential oil/polyurea membrane	(Y. Liu et al., 2021)
	In situ polymerization	Easy industrial production; can be controlled by adjusting the dispersant	Cinnamon oil/ silicon dioxide/poly(melamine formaldehyde)	(Li et al., 2020)
	Microemulsion polymerization	Enhance the protection effect on the core material	Myrcene/octadecane /Poly(N-isopropylacrylamide)	(Wen et al., 2024)

### 2.2 Hydrogel-nanoparticle composite systems

**Fig. 2:**
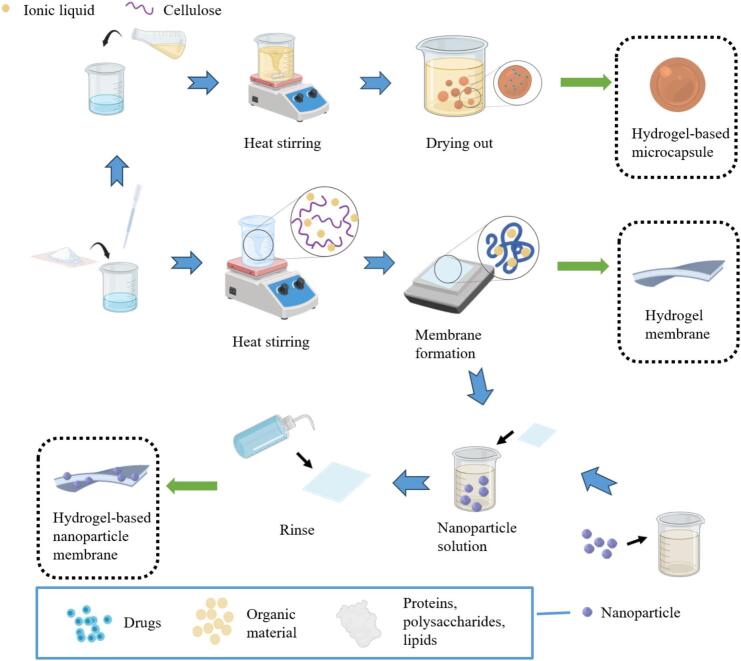
Demonstration of the preparation process of the hydrogel system.

**Fig. 3:**
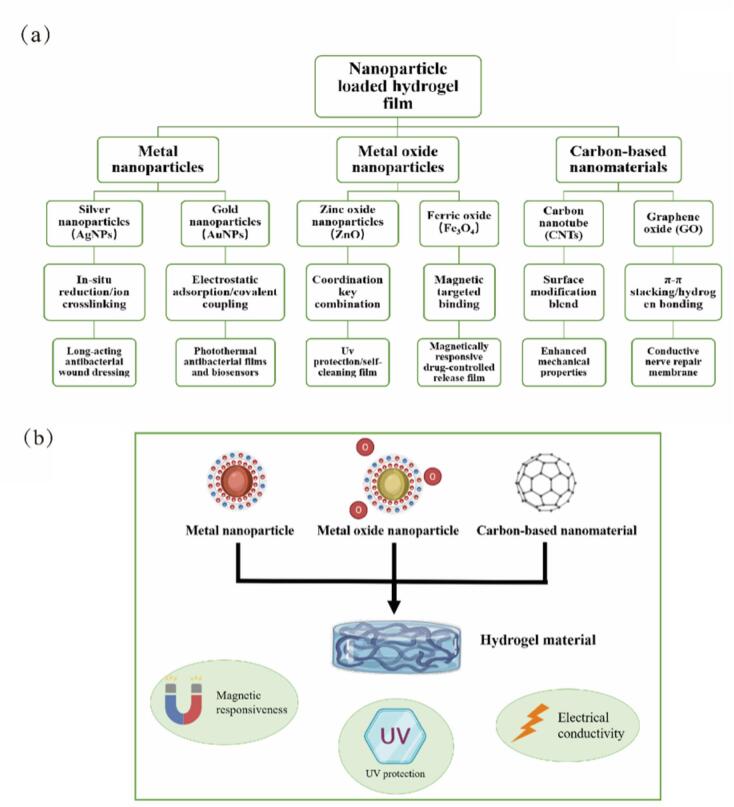
The binding methods of nanoparticles to hydrogel films, including metal nanoparticles, metal oxide nanoparticles and carbon-based nanomaterials.

Nanoparticle-loaded membranes, as a revolutionary composite material, are formed by embedding or attaching various nanoparticles (including metals, oxides, or polymers) into a thin film substrate, as illustrated in Fig. 2. Nanoparticles typically range from 1 nm to several hundred nanometers in size, while microcapsules have diameters from 1 μm to several hundred microns (Sharratt et al., 2021; Sivanathan et al., 2020). Due to their microscopic size, nanoparticles exhibit high specific surface area and quantum size effects, rendering them highly effective in fields such as catalysis, sensing, and antibacterial applications (Kaur et al., 2023). Fig. 3 presents the classification and applications of nanoparticles combined with hydrogel membranes. Nanoparticles can be categorized into three major types: metal nanoparticles, metal oxide nanoparticles, and carbon-based nanomaterials. Each type of nanoparticle can be integrated into hydrogel membranes through specific binding mechanisms and can function in various application fields. The combination of nanoparticles with hydrogel membranes not only enhances the properties of hydrogels but also expands their applications in biomedicine, environmental engineering, and smart materials. For example, metal nanoparticles can provide antibacterial properties, metal oxide nanoparticles can enhance UV protection and magnetic responsiveness, and carbon-based nanomaterials can improve the mechanical properties and electrical conductivity of hydrogels. Through the incorporation of these nanoparticles, hydrogel membranes can serve in a broader range of advanced applications.

Hydrogel-based nanoparticle-loaded films offer a wide range of substrate options, each with unique properties and application scenarios (Table 2). For example, chitosan membranes loaded with curcumin-zein-quercetin-chondroitin sulfate nanoparticles, which were sensitive to ammonia/acetic acid and possessed antioxidant activity, have been used to detect the freshness of fish fillets (Zhang, Yu, et al., 2024). Natural nano-structural melanin nanoparticles (NMPs) extracted from cuttlefish ink can effectively kill *Listeria monocytogenes* when triggered by near-infrared (NIR) radiation, leading to their application in edible antibacterial food packaging (Liang et al., 2023). Furthermore, blending inorganic nanoparticles with organic polymers, such as chitosan/silver nanoparticle grafted films, significantly enhanced the antibacterial properties of nano-scale edible films compared to simple chitosan films (Duong et al., 2023). The advantages of nano-chitosan are notable, as its small particle size provides a larger specific surface area, thereby greatly improving biological activity (Gao & Wu, 2022). These characteristics make nanoparticle-loaded films highly promising for food preservation. The preparation of these composites requires precise control to ensure the uniform distribution and stable attachment of nanoparticles on the substrate, thereby maximizing their functionality. For instance, Quan et al. (2019) demonstrated that bacteria did not survive in biofilm regions where gentamicin-carrying magnetic nanoparticles (MNPs-G) were uniformly distributed through magnetic field exposure for 5 min, and that *Staphylococcus* killing was more effective under these conditions. Holm et al. (2020) found that the spatial distribution of nanoparticles also determined the stability of the Pd-TiO₂ photocatalyst, as non-uniformly distributed nanoparticles tended to sinter, while uniformly distributed nanoparticles remained stable.

**Table 2:** Examples of applications related to supported nanoparticle membranes based on hydrogels or their derivatives.

Classification	Application	Carrier	Nanoparticle	Characteristics	Ref.
Biomedicine	Delivering bioactive substances	Ca-alginate	CA-loaded zein nanoparticles/CA-loaded caseinate nanoparticles	Antibacterial activity of continuous release of calcium	(Ghadimi et al., 2024)
		Chitosan/polyvinyl Alcohol	*Z*-scheme heterojunction g-C_3_N_4_-TiO_2_	Antimicrobial activity，hydrophilicity	(Gao et al., 2024)
	Medical dressing	Sodium alginate/polyvinyl alcohol blend	Boehmite alumina	Thermal stability, flexibility	(Bora et al., 2022)
		Chitosan/cellulose	Sulfur-doped titanium oxide nanoparticles	Swelling capacity	(Aleem et al., 2021)
		chitosan/sodium alginate	AgNPs	Swelling capacity，antioxidant activity	(Qian Li et al., 2024)
	Drug delivery	Sodium alginate	TiO_2_/ZnO nanoparticles/	Tensile resistance	(Toader et al., 2023)
Food Industry	Food preservation	Sodium alginate/carboxymethyl cellulose	CaCO_3_- cinnamaldehyde nanoparticles	UV protection, water resistance, antibacterial activity	(Tan et al., 2024)
		Gelatin/sodium alginate/carvacrol	Zeolitic imidazolate framework-8 (ZIF-8) nanoparticles	Antioxidant activity, thermal stability	(Li et al., 2023)
	Food active packaging	Chitosan	Proanthocyanidins	Antioxidant, antibacterial and oxygen insulation	(Yu et al., 2022)
		Pullulan	Silver nanoparticles	Hydrophilic, light transmittance	(Wypij et al., 2023)
		Cyclodextrin/hydrophobic polydimethylsiloxane	Ultra-small gold nanoparticles	Humidity-responsive antimicrobial function	(Guo et al., 2024)
	Food delivery system	Chitosan	The clove essential oil-loaded zein-NaCas nanoparticles	Water resistance, light blocking, controlled-release and antibacterial properties	(Hua et al., 2021)

Due to their unique physicochemical properties, hydrogel-based nanoparticle-loaded thin membranes have shown extensive application potential in various fields. Films incorporating different nanoparticles not only enhance material properties but also extend applications into food packaging, medical devices, chemical sensors, and beyond. Each nanoparticle type has specific properties, such as antimicrobial activity, thermal stability, antioxidant properties, and moisture retention, that determine their effectiveness in specific applications (Table 2). Complementing these nanoscale enhancements, microencapsulation technology operates at the microscale to provide robust protection and programmable release of sensitive bioactive ingredients, thereby extending the functional capabilities of hydrogel systems into new application domains.

### 2.3 Hydrogel with microcapsules

**Fig. 4:**
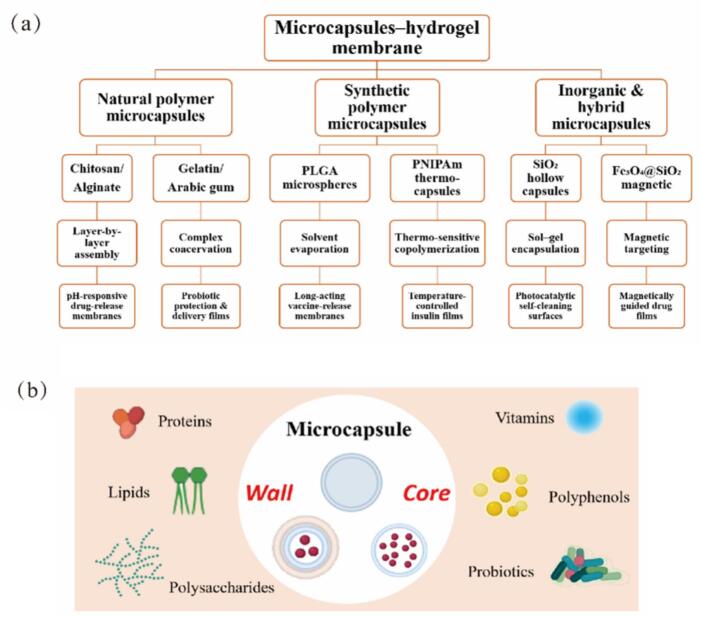
Classification and Examples of microcapsule-hydrogel membranes.

Microencapsulation technology encases solid or liquid active ingredients within tiny capsules using various encapsulation processes. This method creates microcapsules that offer significant stability and controlled release of bioactive substances. The integration of microcapsules with hydrogel systems, along with their applications classified by microencapsulation type and integration method are shown in Fig. 4. In the food industry, microencapsulations are widely used to encapsulate bioactive compounds such as polysaccharides (Li et al., 2024), polyphenols (Larrauri et al., 2023), lutein (Shi et al., 2023), and probiotics with health benefits (Yao et al., 2020; Zhou, Liu, et al., 2022). These encapsulated ingredients remain stable during food processing and release nutrients during digestion, thereby enhancing the nutritional value and functionality of food. The wall materials of microcapsules are typically composed of natural or synthetic polymer materials, including proteins, polysaccharides, and lipids. They not only provide physical and chemical protection for the internal substances, but also realize the precise control of the release rate of the internal substances through their unique structural design. As summarized in Table 1, hydrogel films can be prepared through diverse methods such as chemical crosslinking, free radical polymerization, stepwise polymerization, solution polymerization, radiation polymerization, and physical crosslinking. Each offering distinct advantages in stability, processing speed, adjustability, operational simplicity, reaction mildness, and chemical reagent avoidance. Smart responsive microencapsulation hydrogels prepared by Pickering emulsion polymerization further highlight the versatility of microcapsules (Wen et al., 2024). Their size, shape, and wall thickness can be customized for specific applications. In food packaging, microencapsulated antioxidants (e.g., vitamin E or natural phenolic compounds) combined with hydrogels can create packaging films that slowly release antioxidants. This helps protect food from oxidation and extends its shelf life (Jeong et al., 2021). In drug delivery systems, microencapsulated agents embedded within hydrogels can be designed to release in response to environmental triggers such as pH or temperature changes (Pentela et al., 2018).

As material science and nanotechnology advance, microencapsulation applications in the food industry continue to expand. Hydrogel-microencapsulation systems enhance the nutritional value of food, improve its texture and stability, and enable the development of functional foods with specific health benefits. These technological advancements not only improve food quality and safety but also provide consumers with more diverse and personalized food choices. However, evaluating the mechanical stability and integrity of microencapsulation membranes is crucial for their functionality in applications such as controlled release and cell separation.

## 3 Characterization of hydrogel-based system

### 3.1 Structural characterization (SEM/FTIR/AFM)

**Fig. 5:**
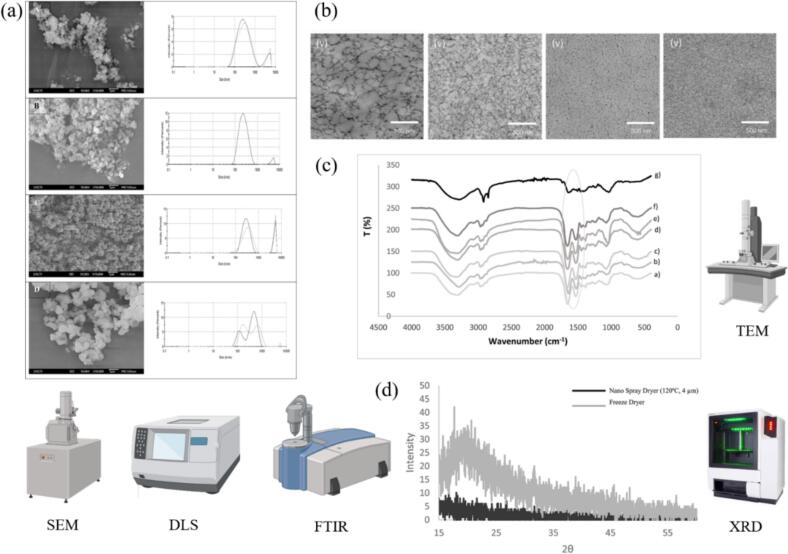
Characterization of different hydrogel samples. (a) SEM micrographs for nanohydrogels dried by spray-drying and freeze-drying. The respective size distribution determined by DLS. (b) GelMA 10 % and HAMA 3 % images from TEM. (c) Fourier infrared transform spectrometry spectra of nanohydrogels dried by spray-drying. (d) XRD patterns of nanohydrogels dried by spray-drying. Reproduced with permission (Aigoin et al., 2025; Bourbon et al., 2020).

Microstructure characterization is crucial for understanding hydrogel membranes' performance in food applications. It offers key insights into mechanical, optical, and thermal properties, which directly influence functionality and effectiveness. For example, investigating microphase separation in hydrogels helped explain mechanical property mechanisms, thereby aiding the development of stronger and more durable hydrogel films for food packaging (Wang, Wang, et al., 2024). In food applications, hydrogel membranes often incorporate active components such as antioxidants, antimicrobial agents, or bioactive substances. Microstructural analysis reveals how these components are dispersed within the hydrogel matrix and interact with it, thus affecting the membrane's performance. Moreover, microstructure characterization is vital for evaluating drug-release behavior and biocompatibility of membrane materials with active functional agents. Fig. 5 comprehensively characterized different hydrogel samples, revealing that nanohydrogel morphology and size distribution varied significantly between spray-drying and freeze-drying methods (SEM and DLS analysis). GelMA 10 % and HAMA 3 % showed distinct structural features (TEM), while FTIR and XRD confirmed the chemical composition and crystallinity differences between the two drying (Aigoin et al., 2025; Bourbon et al., 2020).

Atomic force microscopy (AFM) provides high-resolution imaging of the hydrogel's internal structure, enabling detailed observation of phase separation. This is particularly useful for identifying the distribution of bioactive components, such as hyaluronic acid, which enhances the biocompatibility and functional properties of the membrane (Vu et al., 2023). Scanning electron microscopy (SEM) is widely used to analyze the surface and cross-sectional microstructures of hydrogel membranes, revealing features like pore size, surface roughness, and potential defects. Specifically, SEM analysis revealed pore sizes ranging from 50 to 200 nm in hydrogel film, with porosity directly correlating to water vapor transmission rates, and a denser cross-sectional structure (Zhao et al., 2025). In food packaging applications, SEM helps determine how the hydrogel matrix interacts with embedded nanoparticles or bioactive agents, thereby influencing the membrane's barrier and antimicrobial properties (Ibrahim & Wasfi, 2019). Fourier transform infrared spectroscopy (FTIR) is a powerful tool for analyzing the chemical composition and functional groups within hydrogel membranes. It can determine interactions between the hydrogel matrix and embedded active agents, such as the binding of antioxidants or antimicrobial compounds, and monitor chemical changes under environmental influences, which is crucial for understanding the stability and shelf-life of food packaging materials (Rodriguez et al., 2020). Dynamic light scattering (DLS) measures the size distribution of nanoparticles embedded in hydrogel films, which is essential for understanding how these particles influence the film's mechanical and barrier properties. The particle size and distribution directly affect the release rate of active components, such as antioxidants or antimicrobial agents, potentially extending the shelf-life of food products (Ruiz-Virgen et al., 2024).

In summary, structural characterization is indispensable for optimizing the performance of hydrogel membranes in food applications. Techniques such as AFM, SEM, FTIR, and DLS provide detailed insights into membrane properties and into the distribution and interaction of embedded active agents. This enables the development of advanced hydrogel films with tailored properties for specific food packaging needs, thereby enhancing food safety, shelf-life, and quality. Despite the valuable insights provided by these techniques, a significant limitation is their ex situ and often destructive nature. Consequently, they provide a snapshot of the system under vacuum or dried conditions, failing to capture dynamic structural changes (e.g., pore collapse, component release) in a hydrated state or under real-time storage conditions.

### 3.2 Rheological properties (swelling/rheology)

The swelling behavior of hydrogel films is a critical factor for their performance in food packaging. Swelling capacity, quantified by the swelling rate, reflects a film's ability to absorb and retain water, which is crucial for maintaining the freshness and quality of perishable items (Giz et al., 2020). Conversely, low swelling can enhance barrier properties against oxygen and water vapor, significantly extending shelf-life (Tran et al., 2024). Factors such as solvent chemistry, cross-linking degree, and environmental conditions (e.g., temperature and pH) influence swelling behavior (Kappert et al., 2019). For instance, increasing cross-linking density in hydrogel films can notably reduce their swelling degree, enabling better control of water release and precise humidity regulation (Guo et al., 2020). Furthermore, hydrogel films containing silver-curcumin nanocomposites exhibited higher swelling capacities at lower pH levels compared to pure chitosan hydrogels, highlighting the potential for developing smart packaging materials (Abd El-Hady & Saeed, 2020).

Rheological tests provide insights into how films respond to environmental conditions and mechanical stresses. Parameters such as the elastic and viscous moduli are crucial for assessing mechanical stability (Schmidt et al., 2024). Investigating the rheological properties of polymer solutions, including gelation behavior, viscoelasticity, and their impact on membrane formation, as well as the morphological structure of membranes, is a crucial prerequisite for producing membranes with ideal performance (Mousavi et al., 2023). Rheological properties also significantly impact the filtration efficiency and flux of hydrogel films. Specifically, the viscosity and shear rate of the polymer solution during preparation determine the pore distribution and membrane structure (Kononenko et al., 2017). In nanoparticle-loaded films, the interaction between colloid stability and rheological parameters is noteworthy. For example, gold nanoparticle (GNP)-loaded polymer hydrogel films exhibit dominant elastic properties, and surface modifications of nanoparticles significantly influence the film's rheological behavior (Hwang et al., 2024; Mahmoud et al., 2020). Therefore, the swelling and rheological properties of hydrogel films are critical for their performance in food packaging. Optimizing these properties allows hydrogel films to be tailored to extend the shelf life of perishable foods while ensuring food safety and quality. It is important to note, however, that current rheological and swelling tests are predominantly performed under idealized, static laboratory conditions. Consequently, they fail to replicate the complex and dynamic mechanical stresses (e.g., vibration, impact) or fluctuating temperature and humidity cycles experienced during actual supply chain logistics.

### 3.3 Functional characteristics (antibacterial/hydrophobicity)

Testing the antibacterial properties of materials is essential for assessing their effectiveness in inhibiting microbial growth in applications such as medical devices, food packaging, and personal care products. This testing helps reduce infection risks and enhance product safety. In antimicrobial testing of food packaging systems, a range of common bacteria and microorganisms are typically evaluated, including *Escherichia coli* (Sivaranjani et al., 2022), *Staphylococcus aureus* (Tang et al., 2023), *Pseudomonas aeruginosa* (Selvaraju et al., 2022), *Bacillus subtilis* (Zheng et al., 2023), *Candida albicans* (Mohamed & Madian, 2020), *Aspergillus niger* (Hajinezhad et al., 2020), as well as drug-resistant strains and other specific pathogens. Characterizing antibacterial properties involves various methods, and Table 3 presents the test results of the antibacterial properties of different membrane materials against specific microorganisms. For instance, Qi et al. (2022) incorporated Ag-coated polydopamine (PDA) nanoparticles (PDA@Ag) into a cationic guar gum hydrogel and demonstrated antibacterial effects through photothermal action, using plate coating and SEM for testing. Similarly, Liu et al. (2021) combined the antibacterial ring method with SEM to study ε-polylysine-loaded polyacrylamide and gelatin composite hydrogels. Incorporating nanoparticles with antibacterial properties, such as silver (Ag NPs) and copper nanoparticles (Cu NPs), enhances hydrogel films' antibacterial performance (Fan et al., 2021). The smaller size of nanoparticles provides larger specific surface area, increasing contact with bacteria and improving antibacterial efficacy. For instance, silver nanoparticles' increased surface area-to-volume ratio facilitates ion release, which interacts with bacterial cell membranes, producing free radicals that damage bacterial nucleic acids and proteins, leading to cell death (Liu et al., 2019). However, larger nanoparticles, such as nano-starch particles, can reduce dispersibility and increase contamination risks (Ghanbari et al., 2023).

**Table 3:** Antimicrobial property test of the membranes.

Test method	Membrane	Microbial species	Effectiveness/Effect	Ref.
Disk diffusion method	ZnO/chitosan bio-composite films	*Escherichia coli, Staphylococcus aureus, Klebsiella pneumoniae, Bacillus subtilis*	Antibacterial property effectively improved	(Kalemtas et al., 2022)
	Hydrogel film based on chitosan, lysine, and gelatin	*Escherichia coli，Staphylococcus aureus*	Extended the shelf life of chicken breasts by up to 4 days.	(Li, Jiang, et al., 2025)
	Chitosan-based membranes containing plant extracts	*Staphylococcus aureus, Methicillin resistant Staphylococcus aureus,Escherichia coli, Pseudomonas aeruginosa*	Enhanced antibacterial property by incorporating plant extracts	(Gradinaru et al., 2023)
	Chitosan/peptide films	*Escherichia coli, Bacillus subtilis*	Enhanced antibacterial activity	(Li et al., 2020)
	Silver-Alginate films	*Escherichia coli, Staphylococcus, Aureus, Extended-spectrum beta-lactamases,methicillin-resistant Staphylococcus aureus.*	Inhibition zone increased with AgNO_3_ concentration	(Susilowati et al., 2022)
	Silver nanoparticles-loaded locust bean gum/polyvinyl alcohol hydrogels	*Escherichia coli, Pseudomonas aeruginosa, Staphylococcus aureus, Enterococcus faecalis*	Showed good antibacterial effect	(Matar & Andac, 2020)
Modified AATCC Test Method 100	Nanocellulose hydrogel composited with silver nanoparticles	*Escherichia coli，Staphylococcus aureus*	99.9 %; 99.9 %	(Somsesta et al., 2024)
Liquid culture assay	Egg white protein-based composite films with ε-polylysine	*Escherichia coli, Staphylococcus aureus*	Showed good antibacterial effect	(Li et al., 2023)
Covering method	Sodium alginate/ sodium carboxymethyl cellulose/ collagen edible films	*Staphylococcus aureus*	*Staphylococcus aureus* growth was inhibited at 4 °C for 7 days	(Ma et al., 2019)
Direct contact method	Hydrogels based on gelatin, chitosan and 3-phenyllactic acid	*Staphylococcus aureus, Escherichia coli*	Highly effective antimicrobial property	(Liu et al., 2022)
	Cellulose-based amine films	*Pseudomonas aeruginosa, Staphylococcus aureus, Escherichia coli*	99.99 %; 99.99 %; 99.99 %	(Hamed et al., 2021)

Hydrophobic membranes, in contrast, can emulate certain functional aspects of fruit skins, such as semipermeability and regulated moisture transport. They effectively manage the microenvironment around food, maintaining its freshness and quality (Guo et al., 2019). Hydrophobic coatings prevent fouling and contamination, reducing waste and enhancing consumer experience. Natural lipid additives like beeswax can be used to create biomimetic, superhydrophobic materials for fruit preservation, reducing water transfer rates (Hosseini et al., 2023). Additionally, hydrophobic packaging materials limit oxygen and water penetration, extending food shelf-life. For example, carboxymethyl chitosan/sodium alginate/citric acid-based hydrogel films retained good performance even after multiple regenerations, maintaining strawberry freshness for up to 8 days at 25 °C (Zhang, Guan, et al., 2023). Hydrophobic hydrogel films made from natural polymers, such as plant fibers, improve packaging recyclability and biodegradability, reducing environmental pollution (Ji et al., 2023). The hydrophobicity of these films, commonly assessed by water contact angle, is governed by surface chemical composition and microstructure (Yi et al., 2024). Introducing hydrophobic polymer chains can significantly alter the material's wetness. Micro−/nano-composite structures and low surface energy compositions can achieve superhydrophobicity (Zhang, Kong, et al., 2024). Methods like encapsulation and non-covalent interactions further enhance hydrophobicity (Stephen et al., 2023; Zhai et al., 2020). Thus, both antibacterial properties and hydrophobicity are crucial for hydrogel films in food applications. Antibacterial effects can be enhanced by incorporating nanoparticles with large surface areas, while hydrophobic coatings and structures improve food preservation by regulating the environment and reducing contamination. By tailoring these properties, hydrogel films can effectively extend food shelf life and ensure safety. Perhaps the most critical gap in functional testing is the lack of in-situ, real-time monitoring. Currently, antibacterial activity is measured at endpoints in a petri dish, but it cannot track the real-time kinetics of bacterial inhibition on a packaged food surface. Similarly, the actual humidity regulation around a piece of fruit by a smart hydrogel coating is inferred rather than directly measured in most studies.

## 4 Application in food and quality

### 4.1 Dairy products

Hydrogels are crucial for protecting probiotics in dairy products. The CA-alginate system effectively encapsulates probiotic lactobacilli and yeast, enhancing their survival and stability (Osojnik Crnivec et al., 2021). Similarly, Chu et al. (2025) developed a porous hydrogel using Tremella polysaccharide cross-linked with calcium ions, which protects against damage from stomach acid and bile salts, showing promise for probiotic delivery. Microencapsulation technology further improves probiotic protection and dairy product quality. Kavas et al. (2022) found that adding probiotic microcapsules to goat milk increased organic acids content and dry matter in cheese. Salt-reduced cheese processed with microencapsulated probiotics showed better textural and sensory acceptance during storage and gastrointestinal digestion (Silva et al., 2022). Microencapsulation also enhances probiotic yogurt quality by improving shell materials (Moghaddas Kia et al., 2018). Additionally, Hydrogel-based microcapsules with dual-layer structures enable staged release of active ingredients, optimizing probiotic delivery and improving dairy product functionality.

Hydrogel membrane technology forms a protective barrier on dairy products, preserving their integrity and flavor. Bandyopadhyay et al. (2020) used an essential oil-based hydrogel film to extend “Gouda cheese” shelf-life. In another study, Kashiri et al. (2022) enhanced cheese stability by adding oleic acid to whey protein isolate hydrogel films. Nanoparticle-loaded membranes further boost dairy preservation. Silver and copper nanoparticles in packaging inhibit microbial growth (Ligaj et al., 2020; Lomate et al., 2018), while El-Sayed et al. (2024) demonstrated that oxygen absorbers and antioxidants in films can reduce oxidation and maintain freshness. These technologies collectively enhance dairy product shelf-life and quality. Notably, hydrogels integrate probiotics and enzymes into dairy products, creating novel functional products. For example, β-D-galactosidase immobilized in pectin-based hydrogels helps lactose intolerants by breaking down lactose (Cargnin et al., 2020). Hydrogels can also adjust texture and taste by modifying cross-linking density and pore structure (Oyom et al., 2024). Melo et al. (2020) applied lemongrass essential oil microcapsules in Coalho cheese, improving its nutritional value and sensory quality. Microcapsules can fortify dairy products with nutrients like vitamins and antioxidants (Arab et al., 2019; Danarto, & Rochmadi, & Budhijanto., 2020), thereby improving stability and quality. Pinto et al. (2019) confirmed that microencapsulation technology enhances probiotic survival and yogurt sensory quality. Overall, these technologies significantly improve both the functionality of dairy products and the consumer experience.

### 4.2 Fruits and vegetables

Hydrogel materials are highly effective in passive temperature management due to their thermal response characteristics. Sun et al. (2024) developed a NaCl-containing hydrogel that suppresses temperature rises, protecting peppers from sunburn. The thermochromic properties of hydrogels provide real-time monitoring of fruit storage conditions. Similarly, Shaghaleh et al. (2021) explored stimuli-responsive hydrogels for controlling fruit decay. Furthermore, hydrogel-based microcapsules encapsulate phase change materials (PCMs) to regulate storage temperatures (Xu et al., 2023). These microcapsules protect active ingredients during processing and release them under specific conditions (Ren et al., 2024), offering precise and effective fruit preservation. Jia et al. (2024) studied essential oil microcapsules, such as cinnamon and oregano, for post-harvest fruit and vegetable storage. In addition, the most important function of the hydrogel base film is to maintain the moisture content of food and reduce moisture loss during storage and transportation. At present, a large number of relevant studies have proven its effectiveness in the field of food preservation (Table 4). The CMC/PVA/PEI/TA composite hydrogel film demonstrates excellent water retention ( Yang, Li, et al., 2024). Nanoparticle-loaded films enhance water resistance and impermeability. Bizymis et al. (2023) found that silver nanoparticles addition improve film antibacterial activity, reducing water penetration. Microcapsules encapsulating moisturizers like glycerol or propylene glycol release moisture to reduce water evaporation from fruits (Yang, Guo, et al., 2024). Moreover, Liu, Zhu, et al., 2023 used chlorine dioxide gas microcapsules in PVA films to extend post-harvest shelf-life by regulating respiration and transpiration. These technologies provide comprehensive fresh-keeping solutions for fruits and vegetables.

**Table 4:** Effects of hydrogel-based system for food preservation.

Type	Main component	Preservation mechanism	Applied food	Fresh-keeping effect	Ref.
Hydrogel films	K-carrageenan、carboxymethyl chitosan、kaolin clay	Slow water evaporation, antibacterial, antioxidant	Cherry tomato	Extend shelf life to 9 days, reduce weight loss and maintain fruit firmness	(Yin et al., 2024)
	TEMPO oxidizes nanocellulose、Zinc ion	pH-responsive	Minced pork	Quality testing	(Meng et al., 2023)
Composite film	Polyvinyl alcohol、Ag_2_O-TiO_2_-Bi_2_WO_6_	Inhibit gas exchange and degrade ethylene	Banana	Reduce weightlessness, maintain good appearance color	(Xie et al., 2022)
	Carboxymethyl chitosan、zinc alginate	Water resistant and antimicrobial properties	Chilled meat	Extend the shelf life of pork by 5 days	(Liu et al., 2023)
	Egg albumen、sodium carboxymethylcellulose	Antibacterial property	Meat	Extend shelf life	(Gan et al., 2024)
	Konjac glucomannan、 curdlan	Antibacterial property	Cherry tomato	Reduce decay rate and weight loss rate, maintain soluble solids, titrated acid and vc content	(Chen et al., 2023)
	Gelatin、trehalose	Antibacterial property	Cherry tomato	Prolong storage quality and reduce decay	(Guo et al., 2024)
	Sulfated rice bran polysaccharides、hydroxyethyl cellulose	Reduces polyphenol oxidase activity, increase peroxidase activity	Cherry tomato	Delay deterioration, retain flavor and nutrition	(Liu et al., 2023)
Microcapsule film	*Perilla frutescens* essential oil	Antibacterial and antioxidant	Peach	Extend shelf life	(Tai et al., 2023)
	Gelatin、chitosan、green tea essential oil	Antioxidant property	Strawberry	Delay water loss rate	(Zhao et al., 2023)
	Ethylcellulose	Regulate respiration and transpiration	Litchi	Extend shelf life and maintain sensory properties	(Liu, Zhu, Wang and Huang, 2023)
Hydrogel particles	Alginate、chitosan、curcumin	Antibacterial and antioxidant properties	Omega-3 rich oils	Delay of oxidation	(Hamed et al., 2020)
	Sodium caseinate、highly methyl-esterified pectin、fish oil	Antioxidant property	Low-fat frankfurters	Delay of oxidation	(Salcedo-Sandoval, Cofrades, Ruiz-Capillas, Matalanis, et al., 2015)
	Glucono-β-lactone、anthocyanin、Fmoc-FDFD peptide	pH-responsive	Anthocyanin	Controlled release, delivery	(Li et al., 2024)
	Soybean isolate protein、sodium alginate、blueberry anthocyanins	pH-responsive	High protein drinks	Quality testing	(Ren et al., 2025)
Nanofiber film	Polycaprolactone with D-Cys-loaded mesoporous silica nanomaterials	Antibacterial and antioxidant properties	Fresh pork	Inhibit lipid oxidation and discoloration of fresh pork	(Ye et al., 2025)

Furthermore, hydrogels containing silver nanoparticles effectively inhibit microbial growth, reducing spoilage (Bizymis et al., 2023; Zhang et al., 2022). Some hydrogels block UV radiation, protecting fruit quality (Gong et al., 2024). Loaded nanoparticle membranes further enhance fruit preservation. Tea polyphenols and nano‑silver components offer antioxidant effects and UV shielding (Shao et al., 2018; Zhou et al., 2024). Wu et al. (2023) confirmed that nanomembranes inhibit harmful bacteria like *Escherichia coli*, improving fruit fresh-keeping performance. These technologies ensure food safety and extend shelf life through antimicrobial and antioxidant mechanisms. Notably, hydrogel technology regulates temperature and water retention in fruits and vegetables, extending shelf life. For example, CPP hydrogel delays banana browning and softening, maintaining commercial value (Gang et al., 2024). The multi-layer functional konjac glucomannan/xanthan gum self-healing coating has a significant effect on banana fresh-keeping, which can effectively prolong the shelf life of bananas, reduce post-harvest losses, and improve food safety and quality. Loaded nanoparticle membranes also show potential in extending fruit and vegetable shelf-life by absorbing ethylene gas and delaying ripening (Pang et al., 2024; Wei et al., 2023). Collectively, these advanced technologies help reduce post-harvest losses while enhancing food safety and quality.

### 4.3 Meat products

Hydrogels help preserve meat product freshness by reducing water loss during storage and transportation. Zhang et al. (2020) developed a composite film containing thyme essential oil, extending refrigerated meat shelf-life to ten days. Hydrogels can also be loaded with antimicrobial agents like organic acids and natural extracts, which are released slowly to inhibit microbial growth and enhance food safety (Guo, 2024). Sharma et al. (2021) used maltodextrin and alginate-based food films with *Vibrio albus* to improve meat shelf-life extension. Microencapsulation technology significantly enhances the sensory properties of meat products. Fish oil microcapsules combined with a salt-reduction formulation can increase the omega-3 fatty acid content and improve the taste of hamburger meat (Carlos Solomando et al., 2023). Microencapsulations can also encapsulate microorganisms such as lactic acid bacteria with antibacterial properties, improving probiotic protection and meat product quality (Munekata et al., 2022). This technology can mask undesirable flavors or odors, such as the fishy taste of fish oil (Elsebaie et al., 2022). Microencapsulation protects sensitive ingredients like flavors (Yan et al., 2022), colors (Zeng et al., 2022), and vitamins (Espinoza-Espinoza et al., 2024) from environmental damage. Furthermore, it can simulate fat texture and function in food processing, reducing undesirable texture changes and ensuring flavor stability. Hazelnut oil microcapsules can replace beef burger lipids, improving fatty acid profiles without compromising taste (Alasalvar et al., 2022).

### 4.4 Active substances in food

**Fig. 6:**
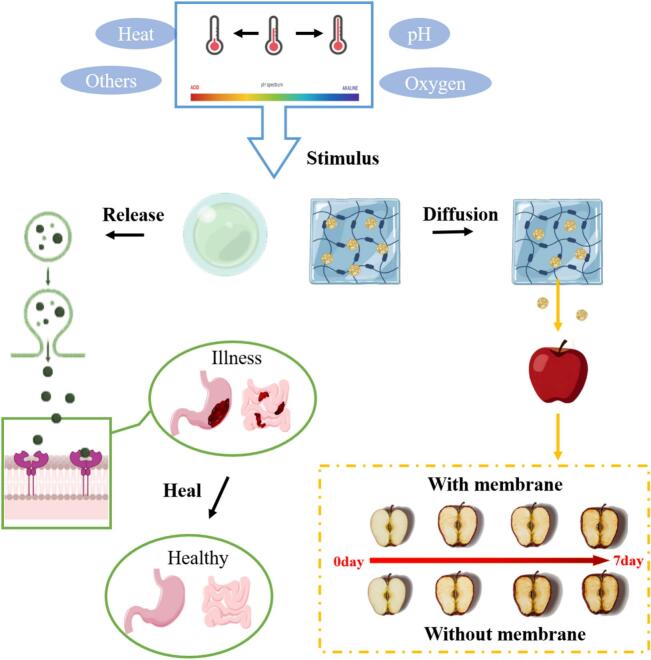
The diffusion and release of hydrogels under different stimuli, and their applications in biomedicine and food preservation.

Delivery systems are essential to ensure the stability and bioavailability of bioactive compounds in various food matrices. Fig. 6 demonstrates how hydrogel microcapsules or hydrogel-loaded nanoparticle membranes respond to external stimuli, and the subsequent process by which active substances diffuse to reach gastrointestinal targets or fruit tissues. These systems are designed to encapsulate polyunsaturated lipids, protecting them in foods, and enhancing their bioavailability in the gastrointestinal tract. For instance, fish oil droplets trapped in casein-rich hydrogel microspheres have been shown to improve lipid oxidation stability, as demonstrated by Zhang et al. (2014). Specifically, n-3 fatty acids encapsulated in filled hydrogel particles not only significantly reduced the oxidation rate compared to oil-in-water (O/W) emulsions but also effectively delayed lipid oxidation in low-fat frankfurters (Salcedo-Sandoval, Cofrades, Ruiz-Capillas, & Jimenez-Colmenero, 2015). Similarly, anthocyanins, which possess excellent biological activity and pH sensitivity, often suffer from poor stability. However, innovative approaches have been developed to enhance their stability. For example, multi-layer hydrogel beads combined with anthocyanins have been utilized to monitor the freshness of pork and chicken breast meat (Li, Chen, et al., 2025). These hydrogel beads effectively improve the stability of anthocyanins. In another study, alginate-based hydrogels combined with ferric ions (SA/PCA/Fe) were reported to enhance the thermal stability of free anthocyanins by embedding them and controlling their release in a simulated gastrointestinal tract (Mao et al., 2023). Furthermore, composite hydrogels are widely used to protect probiotics from harsh environments. For example, *C. crustorum* MN047 embedded in low methoxyl pectin (LMP) and sodium alginate (SAG) composite hydrogels exhibited significantly enhanced viability in a simulated gastrointestinal tract (Zhang, Wang, et al., 2023). Similarly, *Pediococcus pentosaceus* encapsulated in calcium alginate gel beads (CAGBs) showed excellent survival ability under harsh conditions (Mgomi et al., 2024). Overall, these studies highlight the importance and effectiveness of integrated hydrogel delivery systems in enhancing the stability and functionality of bioactive compounds and probiotics in food applications.

## 5 Current challenges and future perspectives

### 5.1 Current challenges and limitations

Despite the significant promise of hydrogel-nanoparticle-microcapsule systems, several critical barriers hinder their widespread commercialization and practical application. Compromised reliability is exhibited by responsive hydrogel membranes, particularly those functionalized with molecularly imprinted polymers or bioreceptors, under real-world conditions. Environmental variables such as temperature fluctuations, changing relative humidity, pH shifts from food metabolites, and cross-reactivity with *co*-existing substances like polyphenols, sulfites, and organic acids can induce signal drift, baseline instability, and false positives or negatives (Guan et al., 2025; Rungsima et al., 2020). For instance, receptor spacing can be altered by swelling-contraction cycles in thermo-responsive hydrogels, leading to misclassification errors in pathogen detection (Niu et al., 2024). Furthermore, current calibration protocols lack standardization across different food matrices such as liquid milk versus solid meat versus respiring fruits, necessitating matrix-specific validation studies.

Profound safety and scalability concerns are revealed by current literature. Manufacturing scalability is regarded as severely limited, as sophisticated synthesis protocols are considered low-yield and energy-intensive, and precise control over temperature, pH, and shear forces is demanded, which is deemed unsuitable for continuous industrial production. Achieving uniform dispersion of nanoparticles (e.g., silver, zinc oxide) without agglomeration and maintaining consistent microencapsulation efficiency across batches remain critical technical hurdles. Moreover, production costs are driven above $50 per kilogram due to reliance on high-purity polymers, making these systems economically uncompetitive with conventional preservatives (Gupta et al., 2024). More alarmingly, nanoparticle migration into food under high-temperature or acidic conditions poses serious health risks. Recent studies demonstrate up to a sevenfold increase in silver release into food substrates (Ren et al., 2023). Ingested silver and silicon dioxide nanoparticles can significantly alter gut microbiome composition, cause intestinal epithelial damage, and may lead to bioaccumulation of undigested colloidal silver and subsequent cellular dysfunction. Furthermore, the environmental fate of these composites is poorly characterized, and soil ecosystems and edible plants are potentially disrupted by nanoparticle release during disposal, yet significant knowledge gaps regarding nanomaterial behavior in waste treatment are acknowledged by current EU legislation. Compounding these issues, substantial market barriers are created by a fragmented global regulatory framework. The EU currently authorises only three nanomaterials for food contact, each requiring case-by-case safety assessment (Peters et al., 2016). Conversely, the U.S. FDA lacks nano-specific regulations, and detection methods are widely considered inadequate (Yemmireddy & Hung, 2017). This regulatory uncertainty, coupled with consumer scepticism toward nanofood, results in most innovations being trapped in the difficulty of commercialization.

### 5.2 Future perspectives and research directions

The future lies in developing low-energy consumption and scalable synthetic routes. Technologies such as photopolymerisation and 3D/4D printing enable rapid, spatially controlled curing and the on-demand manufacturing of complex structures. At the same time, cost and environmental issues can be simultaneously addressed by using agricultural and food processing waste, such as cellulose nanocrystals extracted from fruit peels or chitin from shellfish waste, as low-cost and sustainable raw materials (Markevičiūtė & Varžinskas, 2022; Teixeira-Costa & Andrade, 2021). Unparalleled monodispersity and control in the production of microcapsules and nanoparticles are ensured by the process intensification of microfluidic technology, promoting a smooth transition to industrial-scale production. Future systems must evolve from single-function platforms to integrated, multi-functional platforms capable of performing a range of coordinated actions. Research should focus on creating synergistic combinations, such as encapsulating ethylene-removing nanoparticles (e.g., cerium dioxide) with antioxidant microcapsules for dual-action fruit preservation (Popov et al., 2018). Furthermore, self-healing hydrogels should be developed to repair mechanical damage generated during transportation, thereby maintaining the integrity of the packaging (Wu et al., 2025). These systems can also expand application boundaries by enabling targeted nutrient delivery, for instance, releasing vitamins, minerals, or prebiotics upon consumption to enhance the food's nutritional profile. To ensure safety, a safe-by-design approach is essential. In material selection, priority should be given to Generally Recognized As Safe (GRAS) components; food-grade nanoparticles and biopolymers should be used to minimise toxicological risks, and high-throughput toxicological screening models should be employed to rapidly assess new composites. At the same time, clear, science-based standards and testing protocols must be established for approving new packaging materials, requiring active cooperation with industry and regulators such as the FDA and EFSA.

Systems are enhanced by Artificial Intelligence (AI) integration through the embedding of intelligent capabilities like learning and decision-making into hydrogel technology, accelerating development beyond traditional methods (Li, Gan, et al., 2025). Since hydrogels were defined in 1960 as cross-linked, water-swelling networks, they have evolved into stimuli-responsive smart materials. The intersection with AI began in the 1990s–2000s through computational modelling for predicting hydrogel behaviors (Negut & Bita, 2023). Recent advances now enable automated formulation optimization, high-throughput property screening, and real-time monitoring via integrated sensors, particularly in soft robotics, and significant cost reduction by minimising lab experiments (Duzs et al., 2024). Moreover, AI-driven neurobionic tactile sensing allows hydrogels to simulate biological skin functions (Wang, Yang, et al., 2024). This synergy positions the AI-hydrogel collaboration as a transformative frontier with vast potential for future innovation.

## 6 Conclusion

This review systematically analyses the development, characterization, and application of advanced hydrogel-based systems that integrate nanoparticles and microcapsules for food preservation. The incorporation of nanoparticles (e.g., Ag, Cu, melanin NPs) and microencapsulated bioactive agents (e.g., antioxidants, probiotics, antimicrobials) into hydrogel matrices significantly enhances mechanical stability, antibacterial efficacy, humidity regulation, and stimuli-responsive release. These advances are illustrated across diverse applications. Hydrogel-microencapsulation systems can protect probiotics in dairy products to extend shelf life. Thermo-responsive hydrogels can reduce water loss in fruits and vegetables while enabling freshness monitoring. Essential oil-loaded hydrogels inhibit spoilage in meat products while masking undesirable odors. Despite these advances, significant challenges remain in scalability, safety, and sustainability. Industrial adoption requires low-energy synthesis routes (e.g., photopolymerization) and agricultural waste valorization (e.g., cellulose nanocrystals from fruit peels) to reduce costs. Meanwhile, rigorous toxicological assessments of nanoparticle migration (Ag, SiO₂) and biodegradable designs using marine polymers (alginate, chitosan) are essential to address health and environmental concerns.

To advance the field, future research should prioritise three directions. First, multifunctional synergy, such as combining ethylene-scavenging nanoparticles (CeO₂) with antioxidant microcapsules for dual-action preservation. Second, intelligent packaging systems integrating biosensors for real-time pathogen monitoring with AI-driven optimization of release kinetics under dynamic storage conditions. Third, a “design-safe” strategy should be implemented. This strategy prioritises GRAS components and supports collaboration with regulators (e.g., FDA, EFSA) to establish clearer approval pathways. Ultimately, hydrogel-nanoparticle-microcapsule ternary systems represent a paradigm shift from passive to active food preservation, offering extended shelf-life, enhanced nutrition delivery, and waste reduction. Successful implementation depends on interdisciplinary work across materials science, toxicology, and food engineering. It also requires solutions to scale up and safety constraints. AI-enabled materials design combined with circular-economy thinking is a key frontier. It can support predictive modelling and accelerate the development of next-generation systems. As global food waste approaches 1.3 billion tonnes per year, these technologies align with the UN Sustainable Development Goals, including Zero Hunger and Responsible Consumption and Production. They may help to improve food security while meeting demand for fresh, sustainable products. Future progress will rely on intelligent materials design, robust safety-by-design protocols, and industry–academia–regulator partnerships.

## CRediT authorship contribution statement

**Hui Zhou:** Conceptualization. **Wenying Yuan:** Writing – original draft, Visualization, Methodology, Data curation, Conceptualization. **Chenfeng Xu:** Data curation. **Xuan Huang:** Writing – review & editing. **Weitao Zhao:** Supervision, Methodology. **Zhen Wu:** Methodology. **Daodong Pan:** Conceptualization. **Jie Luo:** Investigation. **Lei Zhou:** Writing – original draft, Methodology, Investigation, Formal analysis, Data curation. **Xiankang Fan:** Project administration, Funding acquisition, Conceptualization.

## Declaration of competing interest

The authors declare that they have no known competing financial interests or personal relationships that could have appeared to influence the work reported in this paper.

## Data Availability

Data will be made available on request.

## References

[bb0005] Abd El-Hady M.M., Saeed S.E.-S. (2020). Antibacterial properties and pH sensitive swelling of Insitu formed silver-curcumin nanocomposite based chitosan hydrogel. Polymers.

[bb0010] Aigoin J., Payré B., Minvielle Moncla J., Escudero M., Goudouneche D., Ferri-Angulo D., Calmon P.-F., Vaysse L., Kemoun P., Malaquin L., Foncy J. (2025). Comparative analysis of Electron microscopy techniques for hydrogel microarchitecture characterization: SEM, Cryo-SEM, ESEM, and TEM. ACS Omega.

[bb0015] Alasalvar H., Kocer Alasalvar G., Yıldırım Z. (2022). Effect of partial fat replacement by hazelnut oil microcapsules in beef burger formulations on physicochemical properties, fatty acid composition, and sensory attributes. Journal of Food Processing and Preservation.

[bib715] Aleem A.R., Shahzadi L., Nasir M., Hajivand P., Alvi F., Akhtar A., Yar M. (2021). Developing sulfur‐doped titanium oxide nanoparticles loaded chitosan/cellulose‐based proangiogenic dressings for chronic ulcer and burn wounds healing. Journal of Biomedical Materials Research Part B: Applied Biomaterials.

[bb0020] Arab M., Razavi S.H., Hosseini S.M., Nayebzadeh K., Meybodi N.M., Khanniri E., Mortazavian A.M. (2019). Production and characterization of functional flavored milk and flavored fermented milk using microencapsulated canthaxanthin. LWT - Food Science and Technology.

[bb0025] Bandyopadhyay S., Saha N., Zandraa O., Pummerová M., Sáha P. (2020). Essential oil based PVP-CMC-BC-GG functional hydrogel sachet for ‘cheese’: Its shelf life confirmed with anthocyanin (isolated from red cabbage) bio stickers. Foods.

[bb0030] Bizymis A.-P., Kalantzi S., Mamma D., Tzia C. (2023). Addition of silver nanoparticles to composite edible films and coatings to enhance their antimicrobial activity and application to cherry preservation. Foods.

[bib714] Bora, D., Jayaramudu, J., Saikia, P., Bohra, R. C., Phukan, L., S, P. S., Ray, S. S., & Sadiku, E. R. (2022). Effect of boehmite alumina nanoparticles on the physical and chemical characteristics of eco-friendly sodium alginate/polyvinyl alcohol bio-nanocomposite film. *International Journal of Polymer Analysis and Characterization, 27*(4), 236-251. 10.1080/1023666x.2022.2061749.

[bb0035] Bourbon A.I., Barbosa-Pereira L., Vicente A.A., Cerqueira M.A., Pastrana L. (2020). Dehydration of protein lactoferrin-glycomacropeptide nanohydrogels. Food Hydrocolloids.

[bb0040] Cargnin M.A., de Souza A.G., de Lima G.F., Gasparin B.C., Rosa D.D.S., Paulino A.T. (2020). Pinus residue/pectin-based composite hydrogels for the immobilization of beta-D-galactosidase. International Journal of Biological Macromolecules.

[bb0045] Carlos Solomando J., Vázquez F., Antequera T., Folgado C., Perez-Palacios T. (2023). Addition of fish oil microcapsules to meat products – Implications for omega-3 enrichment and salt reduction. Journal of Functional Foods.

[bib709] Cetinkaya N., Koc T.B., Karabulut I. (2021). Oxidative Stability and In Vitro Release Properties of Encapsulated Wheat Germ Oil in Saccharomyces cerevisiae Cell‐Based Microcapsules. European Journal of Lipid Science and Technology.

[bib742] Chen K., Tian R., Xu G., Wu K., Liu Y., Jiang F. (2023). Characterizations of konjac glucomannan/curdlan edible coatings and the preservation effect on cherry tomatoes. International Journal of Biological Macromolecules.

[bib704] Chen Y., Wang Z., Dai Y., Yang D., Qiu F., Li Y., Zhang T. (2023). Green Food Packaging with Integrated Functions of High-Efficiency Radiation Cooling and Freshness Monitoring. ACS Sustainable Chemistry & Engineering.

[bb0050] Chu L., Deng Y., Zhang M., Chen J., Lian Y., Chen B., Xie L., Jiang Y. (2025). The characteristics of sodium alginate-tremella polysaccharide assembled hydrogel induced by calcium ion and its protective effect on *lactobacillus rhamnosus*. Food Hydrocolloids.

[bb0055] Chuang E.-Y., Chiang C.-W., Wong P.-C., Chen C.-H., Liu J. (2018). Hydrogels for the application of articular cartilage tissue engineering: A review of hydrogels. Advances in Materials Science and Engineering.

[bb0060] Danarto Y.C., Rochmadi, Budhijanto (2020). Microencapsulation of riboflavin (vitamin B2) using alginate and chitosan : Effect of alginate and chitosan concentration upon encapsulation efficiency. IOP Conference Series: Materials Science and Engineering.

[bb0065] Du L., Huang X., Li Z., Qin Z., Zhang N., Zhai X., Shi J., Zhang J., Shen T., Zhang R., Wang Y. (2025). Application of smart packaging in fruit and vegetable preservation: A review. Foods.

[bib738] Du P., Xu Y., Shi Y., Xu Q., Li S., Gao M. (2022). Preparation and shape change of silver nanoparticles (AgNPs) loaded on the dialdehyde cellulose by in-situ synthesis method. Cellulose (Lond).

[bb0070] Duong Q.X., Do N.H., Pham H.T., Ngo T.H.A. (2023). The enhancement of antibacterial and anti-biofouling properties of chitosan/silver nanoparticles-grafted composite polyamide membrane. Journal of Applied Polymer Science.

[bb0075] Duzs B., Skarsetz O., Fusi G., Lupfer C., Walther A. (2024). Mechano-adaptive meta-gels through synergistic chemical and physical information-processing. Nature Communications.

[bb0080] El Sayed M.M. (2023). Production of polymer hydrogel composites and their applications. Journal of Polymers and the Environment.

[bb0085] El-Sayed S.M., El-Sayed H.S., Hashim A.F., Youssef A.M. (2024). Valorization of edible films based on chitosan/hydroxyethyl cellulose/olive leaf extract and TiO(2)-NPs for preserving sour cream. International Journal of Biological Macromolecules.

[bb0090] Elsebaie E.M., Kassem M.M., Mousa M.M., Basuony M.A.M., Zeima N.M., Essa R.Y. (2022). Cod liver oil’s encapsulation into sodium alginate/lupin protein beads and its application in functional meatballs’ preparation. Foods.

[bb0095] Espinoza-Espinoza L.A., Muñoz-More H.D., Nole-Jaramillo J.M., Ruiz-Flores L.A., Arana-Torres N.M., Moreno-Quispe L.A., Valdiviezo-Marcelo J. (2024). Microencapsulation of vitamins: A review and meta-analysis of coating materials, release and food fortification. Food Research International.

[bib750] Fan X., Yahia L.H., Sacher E. (2021). Antimicrobial Properties of the Ag, Cu Nanoparticle System. Biology.

[bib741] Gan C., Wang J., Yuan Z., Cui M., Sun S., Alharbi M., Yang D.-P. (2024). Polysaccharide- and protein-based edible films combined with microwave technology for meat preservation. International Journal of Biological Macromolecules.

[bb0100] Gang F., Xu M., Zhang S., Zhang C., He J., Xiao Y., Wang H., Liu Z., Sun X., Zhang J. (2024). Biodegradable active composite hydrogel packaging for postharvest climacteric bananas preservation. Food Chemistry.

[bib740] Gao X., Cao L., Wang L., Liu S., Zhang M., Li C., Xu J. (2024). Z-scheme heterojunction g-C3N4-TiO2 reinforced chitosan/poly(vinyl alcohol) film: Efficient and recyclable for fruit packaging. International Journal of Biological Macromolecules.

[bb0105] Gao Y., Wu Y. (2022). Recent advances of chitosan-based nanoparticles for biomedical and biotechnological applications. International Journal of Biological Macromolecules.

[bib713] Ghadimi A.H., Amiri S., Radi M. (2024). Improving the performance of Ca-alginate films through incorporating zein–caseinate nanoparticles-loaded cinnamaldehyde. International Journal of Biological Macromolecules.

[bb0110] Ghanbari R., Heidari A.A., Mahdavi H. (2023). Core-shell antibacterial conjugated nanostarch incorporated PVDF membrane for fast and efficient dye separation. Journal of Environmental Chemical Engineering.

[bb0115] Giz A.S., Berberoglu M., Bener S., Aydelik-Ayazoglu S., Bayraktar H., Alaca B.E., Catalgil-Giz H. (2020). A detailed investigation of the effect of calcium crosslinking and glycerol plasticizing on the physical properties of alginate films. International Journal of Biological Macromolecules.

[bb0120] Gong W., He W.-Y., Hou Y.-Y., Li Y.-X., Hu Y.-Y., Zhu B.-W., Hu J.-N. (2024). Polyvinyl alcohol-based multifunctional hydrogel film: A novel strategy for food preservation packaging. Food Bioscience.

[bib724] Gradinaru L.M., Barbalata-Mandru M., Enache A.A., Rimbu C.M., Badea G.I., Aflori M. (2023). Chitosan Membranes Containing Plant Extracts: Preparation, Characterization and Antimicrobial Properties. International Journal of Molecular Sciences.

[bb0125] Guan Y., Enock K., Su J., Yuan Z., Jin T., Xia Z., Dandan Z. (2025). A high sensitivity and flexibility detection sensor for oxygen concentration based on polyanionic cellulose/locust bean gum/polyacrylamide hydrogel combination. Polymer.

[bb0130] Guo H., Nakajima T., Hourdet D., Marcellan A., Creton C., Hong W., Gong J.P. (2019). Hydrophobic hydrogels: Hydrophobic hydrogels with fruit-like structure and functions. Advanced Materials.

[bib753] Guo H., Xu Y., Chen H., Si X., Zhou M., Zhu E. (2024). Antagonistic yeast and trehalose-enriched gelatin film: A bioactive antifungal packaging film for cherry tomato preservation. Food Packaging and Shelf Life.

[bib721] Guo M., Shen M., Zhu Y., Sogore T., Ding T. (2024). Ultra-small gold nanoparticles embedded cyclodextrin metal-organic framework composite membrane to achieve antibacterial and humidity-responsive functions. Carbohydrate Polymers.

[bb0135] Guo X. (2024). Research progress of antibacterial and antioxidant packaging materials for meat: A review. Journal of Food Process Engineering.

[bb0140] Guo Y., Bae J., Fang Z., Li P., Zhao F., Yu G. (2020). Hydrogels and hydrogel-derived materials for energy and water sustainability. Chemical Reviews.

[bb0145] Gupta R.K., Guha P., Srivastav P.P. (2024). Investigating the toxicological effects of nanomaterials in food packaging associated with human health and the environment. Journal of Hazardous Materials Letters.

[bb0150] Hajinezhad S., Razavizadeh B.M., Niazmand R., Ghasemi I. (2020). Antimicrobial, mechanical, and physicochemical properties of ethylene vinyl alcohol (EVOH) extruded films blended with propolis. International Journal of Food Properties.

[bib731] Hamed O., Radad R., Jodeh S., Deghles A., Qrareya H., Dagdag O., Adwan G. (2021). Design, synthesis and antimicrobial properties of cellulose-based amine film. Polymer Bulletin.

[bib747] Hamed S.F., Hashim A.F., Abdel Hamid, Abd-Elsalam K.A., Golonka I., El-Sherbiny I.M. (2020, Jul 1). Edible alginate/chitosan-based nanocomposite microspheres as delivery vehicles of omega-3 rich oils. Carbohydr Polym.

[bb0155] Holm A., Goodman E.D., Stenlid J.H., Aitbekova A., Zelaya R., Diroll B.T., Cargnello M. (2020). Nanoscale spatial distribution of supported nanoparticles controls activity and stability in powder catalysts for CO oxidation and photocatalytic H2 evolution. Journal of the American Chemical Society.

[bb0160] Hosseini S.F., Mousavi Z., McClements D.J. (2023). Beeswax: A review on the recent progress in the development of superhydrophobic films/coatings and their applications in fruits preservation. Food Chemistry.

[bib706] Hu X., Jia X., Zhi C., Jin Z., Miao M. (2019). Improving the properties of starch-based antimicrobial composite films using ZnO-chitosan nanoparticles. Carbohydrate Polymers.

[bib722] Hua L., Deng J., Wang Z., Wang Y., Chen B., Ma Y., Xu B. (2021). Improving the functionality of chitosan-based packaging films by crosslinking with nanoencapsulated clove essential oil. International Journal of Biological Macromolecules.

[bb0165] Huang Y., Guan Q., Wu Y., Zheng C., Zhong L., Xie W., Chen J., Huang J., Wang Q., Zheng Y. (2025). Microencapsulation of Lactiplantibacillus plantarum BXM2 in bamboo shoot-derived nanocellulose hydrogel to enhance its survivability. Gels.

[bb0170] Hwang U., Moon H., Park J., Jung H.W. (2024). Crosslinking and swelling properties of pH-responsive poly(ethylene glycol)/poly(acrylic acid) interpenetrating polymer network hydrogels. Polymers.

[bb0175] Ibrahim N.I., Wasfi A.S. (2019). A comparative study of polyaniline/MWCNT with polyaniline/SWCNT nanocomposite films synthesized by microwave plasma polymerization. Synthetic Metals.

[bb0180] Jeong H.S., Kim E., Nam C., Choi Y., Lee Y.J., Weitz D.A., Choi C.H. (2021). Hydrogel microcapsules: Hydrogel microcapsules with a thin oil layer: Smart triggered release via diverse stimuli. Advanced Functional Materials.

[bb0185] Ji M., Liu X., Li J., Li F., Man J., Li J., Zhang C., Sun K., Qiu Y. (2023). Comparison of biodegradable chitosan-based composite films reinforced by different micron-sized plant fibers for food packaging. Materials Letters.

[bb0190] Jia S., Zhang H., Qi Q., Yan S., Chen C., Liang L. (2024). Transcriptome analysis of the preservation effect of three essential oil microcapsules on okra. Horticulturae.

[bib723] Kalemtas A., Kocer H.B., Aydin A., Terzioglu P., Aydin G. (2022). Mechanical and antibacterial properties of ZnO/chitosan bio-composite films. Journal of Polymer Engineering.

[bb0195] Kappert E.J., Raaijmakers M.J.T., Tempelman K., Cuperus F.P., Ogieglo W., Benes N.E. (2019). Swelling of 9 polymers commonly employed for solvent-resistant nanofiltration membranes: A comprehensive dataset. Journal of Membrane Science.

[bb0200] Karakus N.R., Turk S., Guney Eskiler G., Syzdykbayev M., Appazov N.O., Ozacar M. (2024). Investigation of tannic acid crosslinked PVA/PEI-based hydrogels as potential wound dressings with self-healing and high antibacterial properties. Gels.

[bb0205] Kashiri M., Maghsoudlou Y., Moayedi A. (2022). Fabrication of active whey protein isolate/oleic acid emulsion based film as a promising bio-material for cheese packaging. Food Science and Technology International.

[bb0210] Kaur J., Bhattu M., Rawat M., Varma R.S., Acevedo R., Shaban M., Singh J. (2023). Facile synthesis of carbon quantum dot/silver nanocomposite and its antimicrobial, catalytic and sensing applications. Environmental Research.

[bb0215] Kavas N., Kavas G., Kınık Ö., Ateş M., Şatır G., Kaplan M. (2022). Effect of probiotic and symbiotic microencapsulation supplementation on the physico-chemical characteristics and organic acid content of goat cheese. Journal of Food Processing and Preservation.

[bb0220] Kononenko N., Nikonenko V., Grande D., Larchet C., Dammak L., Fomenko M., Volfkovich Y. (2017). Porous structure of ion exchange membranes investigated by various techniques. Advances in Colloid and Interface Science.

[bb0225] Lan W., He L., Liu Y. (2018). Preparation and properties of sodium carboxymethyl cellulose/sodium alginate/chitosan composite film. Coatings.

[bb0230] Larrauri M., Asensio C.M., Martín M.P., Quiroga P.R., Grosso N.R., Nepote V. (2023). Soymilk stability increase using polyphenols microcapsules. Journal of Food Science and Technology.

[bb0235] Li B., Chen H., Ma Q., Tang T., Bai Y. (2025). A novel polyvinyl alcohol/chitosan-based anthocyanin electrospun colorimetric film for monitoring chicken breast freshness. Food Analytical Methods.

[bib725] Li C., Pei J., Zhu S., Song Y., Xiong X., Xue F. (2020). Development of Chitosan/Peptide Films: Physical, Antibacterial and Antioxidant Properties. Coatings.

[bib736] Li C., Yang Y., Zhang R., Wang J., Zhong S., Cui X. (2025). Chitosan-gelatin composite hydrogel antibacterial film for food packaging. International Journal of Biological Macromolecules.

[bb0240] Li F., Gan L., Yang X., Tan Z., Shi H., Lai C., Zhang D. (2025). Progress of AI assisted synthesis of polysaccharides-based hydrogel and their applications in biomedical field. International Journal of Biological Macromolecules.

[bb0245] Li Q., Liu M., Liu M., Wang H., Zhao Z. (2024). Preparation, characterization and in vitro digestion of jujube polysaccharide microcapsules. Food and Bioproducts Processing.

[bib751] Li W., Bie Q., Zhang K., Linli F., Yang W., Chen X., Qi Q. (2024, Oct). 30). Regulated anthocyanin release through novel pH-responsive peptide hydrogels in simulated digestive environment. Food Chem X.

[bib752] Li X., Lv J., Niu M., Liu S., Wu Y., Liu J., Wang Y.-M. (2023). Characterization and Antibacterial Properties of Egg White Protein Films Loaded with ε-Polylysine: Evaluation of Their Degradability and Application. Foods.

[bib712] Li Y., Liu J., He X., Kong D., Zhou C., Wu H., Hu Y. (2020). Preparation of Cinnamon Oil‐Loaded Antibacterial Composite Microcapsules by In Situ Polymerization of Pickering Emulsion Templates. Macromolecular Materials and Engineering.

[bib718] Li Y., Shan P., Yu F., Li H., Peng L. (2023). Fabrication and characterization of waste fish scale-derived gelatin/sodium alginate/carvacrol loaded ZIF-8 nanoparticles composite films with sustained antibacterial activity for active food packaging. International Journal of Biological Macromolecules.

[bb0250] Li Z., Jiang L., Liu T., Qin R., Xue J., Li H., Liu Y. (2025). Fabrication of pH-responsive chitosan-based hydrogel beads via electrostatic layer-by-layer assembly for visual monitoring of pork freshness. Food Hydrocolloids.

[bb0255] Liang Y., Zhao Y., Sun H., Dan J., Kang Y., Zhang Q., Su Z., Ni Y., Shi S., Wang J., Zhang W. (2023). Natural melanin nanoparticle-based photothermal film for edible antibacterial food packaging. Food Chemistry.

[bb0260] Ligaj M., Tichoniuk M., Cierpiszewski R., Foltynowicz Z. (2020). Efficiency of novel antimicrobial coating based on Iron nanoparticles for dairy products' packaging. Coatings.

[bb0265] Lin Y., Xu J., Gao X. (2024). Development of antioxidant sodium alginate gel beads encapsulating curcumin/gum Arabic/gelatin microcapsules. Food Hydrocolloids.

[bib743] Liu G., Chen B., Liu H., Wang X., Zhang Y., Wang C., Qiao Y. (2023). Effects of Hydroxyethyl Cellulose and Sulfated Rice Bran Polysaccharide Coating on Quality Maintenance of Cherry Tomatoes during Cold Storage. Foods.

[bb0270] Liu H., Li Z., Zhao Y., Feng Y., Zvyagin A.V., Wang J., Lin Q. (2021). Novel diabetic foot wound dressing based on multifunctional hydrogels with extensive temperature-tolerant, durable, adhesive, and intrinsic antibacterial properties. ACS Applied Materials & Interfaces.

[bib708] Liu J., Chen F., Zhang Q., Xing X., Cui G. (2024). Study on Preparation and Performance of Acid pH-Responsive Intelligent Self-Healing Coating. Polymers.

[bb0275] Liu L., Liang W., Zhang Y., Fu Q. (2023). Nanoencapsulation in polymeric materials: Weaving magical coats for microorganisms. Nano Today.

[bb0280] Liu R., Zhu X., Wang J., Huang C. (2023). Activated release of chlorine dioxide gas from polyvinyl alcohol microcapsule (ethylcellulose/sodium-chlorite) hybrid films for active packaging of litchi during postharvest storage. Postharvest Biology and Technology.

[bib735] Liu W., Kang S., Xue J., Chen S., Yang W., Yan B., Liu D. (2023). Self-assembled carboxymethyl chitosan/zinc alginate composite film with excellent water resistant and antimicrobial properties for chilled meat preservation. International Journal of Biological Macromolecules.

[bb0285] Liu Y., Shi L., Su L., van der Mei H.C., Jutte P.C., Ren Y., Busscher H.J. (2019). Nanotechnology-based antimicrobials and delivery systems for biofilm-infection control. Chemical Society Reviews.

[bib730] Liu Y., Wang R., Wang D., Sun Z., Liu F., Zhang D., Wang D. (2022). Development of a food packaging antibacterial hydrogel based on gelatin, chitosan, and 3-phenyllactic acid for the shelf-life extension of chilled chicken. Food Hydrocolloids.

[bib739] Liu Z., Ma W., Hao Y., Bian J., Zhang Y., Wang H., Wang Y. (2024). Development of antimicrobial and antioxidant film by incorporation of modified protein self-assembled nanoparticles into Pickering emulsion. Food Packaging and Shelf Life.

[bb0290] Lomate G.B., Dandi B., Mishra S. (2018). Development of antimicrobial LDPE/cu nanocomposite food packaging film for extended shelf life of peda. Food Packaging and Shelf Life.

[bb0295] Lopez-Polo J., Monasterio A., Cantero-Lopez P., Osorio F.A. (2021). Combining edible coatings technology and nanoencapsulation for food application: A brief review with an emphasis on nanoliposomes. Food Research International.

[bib729] Ma D., Jiang Y., Ahmed S., Qin W., Liu Y. (2019). Physical and antimicrobial properties of edible films containing Lactococcus lactis. International Journal of Biological Macromolecules.

[bb0300] Ma Y., Yang W., Xia Y., Xue W., Wu H., Li Z., Zhang F., Qiu B., Fu C. (2022). Properties and applications of intelligent packaging indicators for food spoilage. Membranes (Basel).

[bb0305] Mahmoud N.N., Hamed R., Khalil E.A. (2020). Colloidal stability and rheological properties of gold nanoparticle–loaded polymeric hydrogels: Impact of nanoparticle’s shape, surface modification, and concentration. Colloid and Polymer Science.

[bb0310] Mao S., Ren Y., Chen S., Liu D., Ye X., Tian J. (2023). Development and characterization of pH responsive sodium alginate hydrogel containing metal-phenolic network for anthocyanin delivery. Carbohydrate Polymers.

[bb0315] Markevičiūtė Z., Varžinskas V. (2022). Plant-origin feedstock applications in fully green food packaging: The potential for tree-free paper and plant-origin bio-plastics in the Baltic Sea region. Sustainability.

[bib727] Matar G.H., Andac M. (2020). Antibacterial efficiency of silver nanoparticles-loaded locust bean gum/polyvinyl alcohol hydrogels. Polymer Bulletin.

[bb0320] Melo A.M.D., Turola Barbi R.C., Souza W.F.C.D., Luna L.C., Souza H.J.B., Lucena G.L., Sousa S. (2020). Microencapsulated lemongrass (*Cymbopogon flexuosus*) essential oil: A new source of natural additive applied to Coalho cheese. Journal of Food Processing and Preservation.

[bib733] Meng X., Shen Q., Song T., Zhao H., Zhang Y., Ren A., Yang W. (2023, Jul). 5). Facile Fabrication of Anthocyanin-Nanocellulose Hydrogel Indicator Label for Intelligent Evaluation of Minced Pork Freshness. Foods.

[bb0325] Merino D., Quilez-Molina A.I., Perotto G., Bassani A., Spigno G., Athanassiou A. (2022). A second life for fruit and vegetable waste: A review on bioplastic films and coatings for potential food protection applications. Green Chemistry.

[bb0330] Mgomi F.C., Yuan L., Farooq R., Lu C.-L., Yang Z.-Q. (2024). Survivability and characterization of the biofilm-like probiotic *Pediococcus pentosaceus* encapsulated in calcium alginate gel beads. Food Hydrocolloids.

[bb0335] Moghaddas Kia E., Ghasempour Z., Ghanbari S., Pirmohammadi R., Ehsani A. (2018). Development of probiotic yogurt by incorporation of milk protein concentrate (MPC) and microencapsulated *lactobacillus paracasei* in gellan-caseinate mixture. British Food Journal.

[bb0340] Mohamed N., Madian N.G. (2020). Evaluation of the mechanical, physical and antimicrobial properties of chitosan thin films doped with greenly synthesized silver nanoparticles. Materials Today Communications.

[bb0345] Mohammed A.E., Abdalhalim L.R., Atalla K.M., Mohdaly A.A.A., Ramadan M.F., Abdelaliem Y.F. (2023). Chitosan and sodium alginate nanoparticles synthesis and its application in food preservation. Rendiconti Lincei. Scienze Fisiche e Naturali.

[bb0350] Mousavi S.M., Raveshiyan S., Amini Y., Zadhoush A. (2023). A critical review with emphasis on the rheological behavior and properties of polymer solutions and their role in membrane formation, morphology, and performance. Advances in Colloid and Interface Science.

[bb0355] Munekata P.E.S., Pateiro M., Tomasevic I., Domínguez R., da Silva Barretto A.C., Santos E.M., Lorenzo J.M. (2022). Functional fermented meat products with probiotics—A review. Journal of Applied Microbiology.

[bib711] Napiorkowska A., Szpicer A., Gorska-Horczyczak E., Kurek M.A. (2024, Apr). 27). Microencapsulation of Essential Oils Using Faba Bean Protein and Chia Seed Polysaccharides via Complex Coacervation Method. Molecules.

[bb0360] Negut I., Bita B. (2023). Exploring the potential of artificial intelligence for hydrogel development—A short review. Gels.

[bb0365] Niu H., Zhang M., Shen D., Mujumdar A.S., Ma Y. (2024). Sensing materials for fresh food quality deterioration measurement: A review of research progress and application in supply chain. Critical Reviews in Food Science and Nutrition.

[bib703] Noor F., Mahmood A., Zafar N., Sarfraz R.M., Rehman U., Ijaz H., Benguerba Y. (2023). Fabrication of pH-responsive hydrogels of perindopril erbumine using black seed extract and β-cyclodextrin co-polymerized with methacrylic acid and methylene bisacrylamide. Journal of Drug Delivery Science and Technology.

[bb0370] Osojnik Crnivec I.G., Neresyan T., Gatina Y., Kolmanic Bucar V., Skrt M., Dogsa I., Poklar Ulrih N. (2021). Polysaccharide hydrogels for the protection of dairy-related microorganisms in adverse environmental conditions. Molecules.

[bb0375] Oyom W., Awuku R.B., Faraji H., Bi Y., Tahergorabi R. (2024). Protein hydrogel formation from chicken processing by-products: Exploring applications in food. Food Research International.

[bb0380] Pang X., Huang Y., Xiao N., Wang Q., Feng B., Ali Shad M. (2024). Effect of EVA film and chitosan coating on quality and physicochemical characteristics of mango fruit during postharvest storage. Food Chemistry: X.

[bb0385] Pentela N., Duraipandy N., Sainath N., Parandhaman T., Kiran M.S., Das S.K., Samanta D. (2018). Microcapsules from diverse polyfunctional materials: Synergistic interactions for a sharp response to pH changes. New Journal of Chemistry.

[bb0390] Peters R.J.B., Bouwmeester H., Gottardo S., Amenta V., Arena M., Brandhoff P., Aschberger K. (2016). Nanomaterials for products and application in agriculture, feed and food. Trends in Food Science & Technology.

[bb0395] Pink D.L., Loruthai O., Ziolek R.M., Wasutrasawat P., Terry A.E., Lawrence M.J., Lorenz C.D. (2019). On the structure of solid lipid nanoparticles. Small.

[bb0400] Pinto S.S., Fritzen-Freire C.B., Dias C.O., Amboni R.D.M.C. (2019). A potential technological application of probiotic microcapsules in lactose-free Greek-style yoghurt. International Dairy Journal.

[bb0405] Popov A.L., Popova N.R., Tarakina N.V., Ivanova O.S., Ermakov A.M., Ivanov V.K., Sukhorukov G.B. (2018). Intracellular delivery of antioxidant CeO(2) nanoparticles via polyelectrolyte microcapsules. ACS Biomaterials Science & Engineering.

[bb0410] Qi X., Huang Y., You S., Xiang Y., Cai E., Mao R., Pan W., Tong X., Dong W., Ye F., Shen J. (2022). Engineering robust ag-decorated polydopamine Nano-photothermal platforms to combat bacterial infection and prompt wound healing. Advanced Science.

[bb0415] Quan K., Zhang Z., Ren Y., Busscher H.J., van der Mei H.C., Peterson B.W. (2019). Homogeneous distribution of magnetic, antimicrobial-carrying nanoparticles through an infectious biofilm enhances biofilm-killing efficacy. ACS Biomaterials Science & Engineering.

[bb0420] Ren L., Liu S., Zhong J., Zhang L. (2024). Revolutionizing targeting precision: Microfluidics-enabled smart microcapsules for tailored delivery and controlled release. Lab on a Chip.

[bb0425] Ren Q., Ma J., Li X., Meng Q., Wu S., Xie Y., Qi Y., Liu S., Chen R. (2023). Intestinal toxicity of metal nanoparticles: Silver nanoparticles disorder the intestinal immune microenvironment. ACS Applied Materials & Interfaces.

[bib748] Ren Y., Wang Y., Yang X., Li L. (2025). May 30). Edible blueberry anthocyanin-loaded soybean protein nanofibers/sodium alginate hydrogel beads: Freshness detection of high protein drinks. Food Chem.

[bb0430] Rodriguez A.K., Mansoor B., Ayoub G., Colin X., Benzerga A.A. (2020). Effect of UV-aging on the mechanical and fracture behavior of low density polyethylene. Polymer Degradation and Stability.

[bb0435] Ruiz-Virgen L., Hernandez-Martinez M.A., Martínez-Mejía G., Caro-Briones R., Herbert-Pucheta E., Río J.M.D., Corea M. (2024). Analysis of structural changes of pH–Thermo-responsive nanoparticles in polymeric hydrogels. Gels.

[bb0440] Rungsima C., Boonyan N., Klorvan M., Kusuktham B. (2020). Hydrogel sensors with pH sensitivity. Polymer Bulletin.

[bb0445] Salcedo-Sandoval L., Cofrades S., Ruiz-Capillas C., Jimenez-Colmenero F. (2015). Filled hydrogel particles as a delivery system for n-3 long chain PUFA in low-fat frankfurters: Consequences for product characteristics with special reference to lipid oxidation. Meat Science.

[bb0450] Salcedo-Sandoval L., Cofrades S., Ruiz-Capillas C., Matalanis A., McClements D.J., Decker E.A., Jimenez-Colmenero F. (2015). Oxidative stability of n-3 fatty acids encapsulated in filled hydrogel particles and of pork meat systems containing them. Food Chemistry.

[bb0455] Schmidt R.F., Kiefer H., Dalgliesh R., Gradzielski M., Netz R.R. (2024). Nanoscopic interfacial hydrogel viscoelasticity revealed from comparison of macroscopic and microscopic rheology. Nano Letters.

[bb0460] Selvaraju N., Ganesh P.S., Palrasu V., Venugopal G., Mariappan V. (2022). Evaluation of antimicrobial and antibiofilm activity of *Citrus medica* fruit juice based carbon dots against *Pseudomonas aeruginosa*. ACS Omega.

[bb0465] Shaghaleh H., Hamoud Y.A., Xu X., Liu H., Wang S., Sheteiwy M., Zhang S. (2021). Thermo-/pH-responsive preservative delivery based on TEMPO cellulose nanofiber/cationic copolymer hydrogel film in fruit packaging. International Journal of Biological Macromolecules.

[bb0470] Shao P., Niu B., Chen H., Sun P. (2018). Fabrication and characterization of tea polyphenols loaded pullulan-CMC electrospun nanofiber for fruit preservation. International Journal of Biological Macromolecules.

[bb0475] Sharma R., Bhat Z.F., Kumar A., Kumar S., Bekhit A.E.D.A., Naqvi Z. (2021). Characterization of Commiphora wightii based bioactive edible film and its efficacy for improving the storage quality of meat products. Journal of Food Safety.

[bb0480] Sharratt W.N., Lee V.E., Priestley R.D., Cabral J.T. (2021). Precision polymer particles by flash nanoprecipitation and microfluidic droplet extraction. ACS Applied Polymer Materials.

[bib737] Shi C., Yang F., Hu L., Wang H., Wang Y., Wang Z., Chen J. (2022). Construction of polysaccharide based physically crosslinked double-network antibacterial hydrogel. Materials Letters.

[bb0485] Shi Z., Kong G., Wang F., Gao H., Wei A., Ren S., Yan X. (2023). Improvement in the stability and bioavailability of pumpkin lutein using β-cyclodextrin microcapsules. Food Science & Nutrition.

[bib701] Siddiqua A., Ranjha N.M., Rehman S., Shoukat H., Ramzan N., Sultana H. (2021). Preparation and characterization of methylene bisacrylamide crosslinked pectin/acrylamide hydrogels. Polymer Bulletin.

[bb0490] Silva R., Pimentel T.C., de Matos E., Junior F., Esmerino E.A., Freitas M.Q., Cruz A.G. (2022). Microencapsulation with spray-chilling as an innovative strategy for probiotic low sodium requeijão cremoso processed cheese processing. Food Bioscience.

[bb0495] Sivanathan A., Dou Q., Wang Y., Li Y., Corker J., Zhou Y., Fan M. (2020). Phase change materials for building construction: An overview of nano−/micro-encapsulation. Nanotechnology Reviews.

[bb0500] Sivaranjani M., McCarthy M.C., Sniatynski M.K., Wu L., Dillon J.-A.R., Rubin J.E., White A.P. (2022). Biofilm formation and antimicrobial susceptibility of E. Coli associated with colibacillosis outbreaks in broiler chickens from Saskatchewan. Frontiers in Microbiology.

[bib728] Somsesta N., Jinnapat A., Fakpiam S., Suksanguan C., Wongsan V., Ouneam W., Hongrattanavichit I. (2024). Antimicrobial and biodegradable hydrogel based on nanocellulose/alginate incorporated with silver nanoparticles as active packaging for poultry products. Scientific Reports.

[bb0505] Stephen K., Gayathri V., Lobo N.P., Phani Kumar B.V.N., Jaisankar S.N., Samanta D. (2023). Improving hydrophobicity of collagen with silica nanoparticles: Probing a noncovalent approach. Langmuir.

[bb0510] Sun L., Sun D.W., Xu L., Tian Y., Zhu Z. (2024). Tunable thermoresponsive hydrogels for temperature regulation and warning in fruit and vegetables preservation. Food Chemistry.

[bb0515] Sun Z., Song C., Wang C., Hu Y., Wu J. (2020). Hydrogel-based controlled drug delivery for Cancer treatment: A review. Molecular Pharmaceutics.

[bib726] Susilowati E., Mahardiani L., Hardini R.D. (2022). The effect of silver nanoparticles toward properties and antibacterial activity of silver-alginate nanocomposite films. Frontiers in Sustainable Food Systems.

[bib744] Tai Z., Zheng M., Yang Y., Xie C., Li Z., Xu C. (2023). Temperature controlled microcapsule loaded with Perilla essential oil and its application in preservation of peaches. Frontiers in Nutrition.

[bib717] Tan X., Zhang S., Liu J., Xiang W., Zhang Q., Tang J. (2024). Effect of Lactiplantibacillus plantarum cell-free supernatant on the physiology, quorum sensing, transcription, and enhanced GABA production of Enterococcus faecium. Lwt.

[bb0520] Tang Y., Zou F., Chen C., Zhang Y., Shen Z., Liu Y., Deng Q., Yu Z., Wen Z. (2023). Antibacterial and antibiofilm activities of Sertindole and its antibacterial mechanism against *Staphylococcus aureus*. ACS Omega.

[bb0525] Teixeira-Costa B.E., Andrade C.T. (2021). Chitosan as a valuable biomolecule from seafood industry waste in the Design of Green Food Packaging. Biomolecules.

[bib702] Tian B., Wang J., Liu Q., Liu Y., Chen D. (2021). Formation chitosan-based hydrogel film containing silicon for hops β-acids release as potential food packaging material. International Journal of Biological Macromolecules.

[bib716] Toader G., Podaru A.I., Diacon A., Rusen E., Mocanu A., Brincoveanu O., Istrate M. (2023). Nanocomposite Hydrogel Films Based on Sequential Interpenetrating Polymeric Networks as Drug Delivery Platforms. Polymers.

[bb0530] Tran K.Q., Nguyen C.T.K., Duong Q.X., Tran T.T.T., Ngo T.H.A. (2024). Development of a chitosan-based food packaging film with the incorporation of pectin and silver nanoparticles for improved antibacterial and physical properties. ChemistrySelect.

[bb0535] Vu T.T., Gulfam M., Jo S.-H., Rizwan A., Joo S.-B., Lee B., Lim K.T. (2023). The effect of molecular weight and chemical structure of cross-linkers on the properties of redox-responsive hyaluronic acid hydrogels. International Journal of Biological Macromolecules.

[bib710] Wang F., Mutukumira A.N. (2022). Microencapsulation of Limosilactobacillus reuteri DPC16 by spray drying using different encapsulation wall materials. Journal of Food Processing and Preservation.

[bb0540] Wang S., Yang T., Zhang D., Hua Q., Zhao Y. (2024). Unveiling gating behavior in Piezoionic effect: Toward Neuromimetic tactile sensing. Advanced Materials.

[bb0545] Wang W., Wang K., Cheng Y., Wu C., Wu R., Huang J., Lai Y. (2024). Bidirectional temperature-responsive thermochromic hydrogels with adjustable light transmission interval for smart windows. Advanced Functional Materials.

[bb0550] Wei H., Li L., Zhang T., Seidi F., Chen Q., Xiao H. (2023). Surface-modified CeO2-octahedron-supported pt nanoparticles as ethylene scavengers for fruit preservation. ACS Applied Nano Materials.

[bb0555] Wen Y., Yu S., Ge Z., Jiang J. (2024). Temperature-responsive microcapsule hydrogel fabricated by Pickering emulsion polymerization for pheromones application. Colloids and Surfaces A: Physicochemical and Engineering Aspects.

[bb0560] Wu W., Zhong J., Yang Z., Qin Z., Shen C., Cai Z., Yang X., Li Q., Wang D., Li J., Li X., Wu D., Chen K. (2025). Preparation of ZnSO4 cross-linked double-network hydrogels via synergistic ionic and Hofmeister effects as in-situ fruit packaging integrating cushioning and monitoring. Food Packaging and Shelf Life.

[bb0565] Wu X., Liu Z., He S., Liu J., Shao W. (2023). Development of an edible food packaging gelatin/zein based nanofiber film for the shelf-life extension of strawberries. Food Chemistry.

[bib720] Wypij M., Rai M., Zemljič L.F., Bračič M., Hribernik S., Golińska P. (2023). Pullulan-based films impregnated with silver nanoparticles from the Fusarium culmorum strain JTW1 for potential applications in the food industry and medicine. Frontiers in Bioengineering and Biotechnology.

[bib734] Xie J., Wang R., Li Y., Ni Z., Situ W., Ye S., Song X. (2022). A novel Ag2O-TiO2-Bi2WO6/polyvinyl alcohol composite film with ethylene photocatalytic degradation performance towards banana preservation. Food Chemistry.

[bb0570] Xu X., Chen H., Wang Q., Su C., Sun Y., Qiu C., Pang J. (2025). Research progress and future trends in smart response packaging for food preservation. Journal of Stored Products Research.

[bb0575] Xu Y., Dong M., Xiao H., Young Quek S., Ogawa Y., Ma G., Zhang C. (2023). Advances in spray-dried probiotic microcapsules for targeted delivery: A review. Critical Reviews in Food Science and Nutrition.

[bb0580] Yan C., Kim S.-R., Ruiz D.R., Farmer J.R. (2022). Microencapsulation for food applications: A review. ACS Applied Bio Materials.

[bb0585] Yang D. (2022). Recent advances in hydrogels. Chemistry of Materials.

[bb0590] Yang H., Li L., Li C., Xu Z., Tao Y., Lu J., Xia X., Tan M., Du J., Wang H. (2024). Multifunctional and antimicrobial carboxymethyl cellulose-based active hydrogel film for fruits packaging and preservation. Food Bioscience.

[bb0595] Yang Z., Guo Y., Zeng C., Sun F., Wang Z., Zhang W., Tian T., Shan L., Zeng Y., Huang Z., Jiang L. (2024). Encapsulation and characterization of ω-3 medium- and long-chain triacylglycerols microencapsulated with different proteins as wall materials. Food Chemistry: X.

[bb0600] Yao M., Xie J., Du H., McClements D.J., Xiao H., Li L. (2020). Progress in microencapsulation of probiotics: A review. Comprehensive Reviews in Food Science and Food Safety.

[bib749] Ye Z., Yuan H., Zhang J., Yan W., Xing C. (2025). D@MSNs-P/PCL antibacterial nanofibers combined with DBD cold plasma for fresh pork preservation. Food Control.

[bb0605] Yemmireddy V.K., Hung Y.C. (2017). Using photocatalyst metal oxides as antimicrobial surface coatings to ensure food safety-opportunities and challenges. Comprehensive Reviews in Food Science and Food Safety.

[bb0610] Yi B., Li T., Yang B., Chen S., Zhao J., Zhao P., Zhang K., Wang Y., Wang Z., Bian L. (2024). Surface hydrophobization of hydrogels via interface dynamics-induced network reconfiguration. Nature Communications.

[bib732] Yin C., Sun Z., Yang Y., Cui M., Zheng J., Zhang Y. (2024). Rapid in situ formation of κ-carrageenan-carboxymethyl chitosan-kaolin clay hydrogel films enriched with arbutin for enhanced preservation of cherry tomatoes. International Journal of Biological Macromolecules.

[bib719] Yu Z., Jiang Q., Yu D., Dong J., Xu Y., Xia W. (2022). Physical, antioxidant, and preservation properties of chitosan film doped with proanthocyanidins-loaded nanoparticles. Food Hydrocolloids.

[bib707] Zang C., Zhang Y., Yang W., Hu Y. (2024). Polycaprolactone/chitosan electrospun nanofibrous membranes loaded Chinese yam polysaccharide for active food packaging. Lwt.

[bb0615] Zeng Y., Wang Y., Tang J., Zhang H., Dai J., Li S., Yan J., Qin W., Liu Y. (2022). Preparation of sodium alginate/konjac glucomannan active films containing lycopene microcapsules and the effects of these films on sweet cherry preservation. International Journal of Biological Macromolecules.

[bb0620] Zhai Z., Xu P., Yao J., Li R., Gong L., Yin Y., Lin Z. (2020). Erythrocyte-mimicking paclitaxel nanoparticles for improving biodistributions of hydrophobic drugs to enhance antitumor efficacy. Drug Delivery.

[bb0625] Zhang F., Wang R., Zhang L., Yan L., Jia Y., Yang J., Wang X., Lü X. (2023). Enhanced viability of probiotics in composite hydrogel beads. Journal of Food Engineering.

[bb0630] Zhang H., Guan G., Lou T., Wang X. (2023). High performance, cost-effective and ecofriendly flocculant synthesized by grafting carboxymethyl cellulose and alginate with itaconic acid. International Journal of Biological Macromolecules.

[bb0635] Zhang L., Chen D., Yu D., Regenstein J.M., Jiang Q., Dong J., Xia W. (2022). Modulating physicochemical, antimicrobial and release properties of chitosan/zein bilayer films with curcumin/nisin-loaded pectin nanoparticles. Food Hydrocolloids.

[bb0640] Zhang L., Yu D., Gu Y., Xu Y., Jiang Q., Yang F., Xia W. (2024). Green halochromic smart and active packaging materials based on chitosan film loading nanoparticles: Functionality, physicochemical properties and application. Food Hydrocolloids.

[bb0645] Zhang Q., Kong B., Liu H., Du X., Sun F., Xia X. (2024). Nanoscale Pickering emulsion food preservative films/coatings: Compositions, preparations, influencing factors, and applications. Comprehensive Reviews in Food Science and Food Safety.

[bb0650] Zhang Y., Zhao W., Lin Z., Tang Z., Lin B. (2023). Carboxymethyl chitosan/sodium alginate hydrogel films with good biocompatibility and reproducibility by in situ ultra-fast crosslinking for efficient preservation of strawberry. Carbohydrate Polymers.

[bb0655] Zhang Y., Zhou L., Zhang C., Show P.L., Du A., Fu J., Ashokkumar V. (2020). Preparation and characterization of curdlan/polyvinyl alcohol/ thyme essential oil blending film and its application to chilled meat preservation. Carbohydrate Polymers.

[bb0660] Zhang Z., Cheng M., Gabriel M.S., Teixeira Neto A.A., da Silva Bernardes J., Berry R., Tam K.C. (2019). Polymeric hollow microcapsules (PHM) via cellulose nanocrystal stabilized Pickering emulsion polymerization. Journal of Colloid and Interface Science.

[bb0665] Zhang Z., Decker E.A., McClements D.J. (2014). Encapsulation, protection, and release of polyunsaturated lipids using biopolymer-based hydrogel particles. Food Research International.

[bb0670] Zhang Z., Yao A., Raffa P. (2024). Transparent, highly stretchable, self-healing, adhesive, freezing-tolerant, and swelling-resistant multifunctional hydrogels for underwater motion detection and information transmission. Advanced Functional Materials.

[bb0675] Zhao M., Yang H., Ran C., Chen N., Liao Z., Hu B., Ji H., Dong J., Sun J. (2025). Chitosan-dialdehyde starch/polyvinyl alcohol double-crosslinked network hydrogel film for degradable food packaging: Addressing mono-crosslinking limitations. Progress in Organic Coatings.

[bib745] Zhao W., Yu L., Gu Y., Ma W., Wang K., Liang J., Liu Q. (2023). Preparation of dual-functional packaging films containing green tea essential oil microcapsules for strawberry preservation: Excellent barrier properties and antioxidant activity. Food Bioscience.

[bb0680] Zhao X., Zou D., Liu Y., Xia Y., Tao J., Zeng Q., Hou Y., Liu M. (2024). Electrospun polylactic acid nanofibers membrane with copper ion-loaded clay nanotubes for fresh-keeping packaging. International Journal of Biological Macromolecules.

[bb0685] Zheng M., Zhu Y., Zhuang Y., Tan K.B., Chen J. (2023). Effects of grape seed extract on the properties of pullulan polysaccharide/xanthan gum active films for apple preservation. International Journal of Biological Macromolecules.

[bb0690] Zhou L., Zhang W., Wang J. (2022). Recent advances in the study of modified cellulose in meat products: Modification method of cellulose, meat quality improvement and safety concern. Trends in Food Science & Technology.

[bb0695] Zhou S., Peng H., Zhao A., Yang X., Lin D. (2024). Konjac glucomannan-based highly antibacterial active films loaded with thyme essential oil through bacterial cellulose nanofibers/Ag nanoparticles stabilized Pickering emulsions. International Journal of Biological Macromolecules.

[bb0700] Zhou T., Liu M., Pan J., Ren J., Tang F., Dai J., Xue F., Ji D. (2022). Combined therapy of probiotic microcapsules and Bomidin in *Vibrio parahaemolyticus*–infected rats. Life.

